# Downregulation of N6-methyladenosine-modified LINC00641 promotes EMT, but provides a ferroptotic vulnerability in lung cancer

**DOI:** 10.1038/s41419-023-05880-3

**Published:** 2023-06-13

**Authors:** Shu Xi, Dao-Jing Ming, Jin-Hui Zhang, Meng-Meng Guo, Shuang-Ying Wang, Yi Cai, Meng-Yang Liu, Dan-Qi Wang, Yi-Jie Zhang, Yafei Li, Shuai Yuan

**Affiliations:** 1 Center for Evidence-Based and Translational Medicine, Zhongnan Hospital of Wuhan University, Wuhan, China; 2 School of Clinical Medicine, Henan University, Kaifeng, China; 3 Department of Respiratory, Huaihe Hospital of Henan University, Kaifeng, China; 4 Department of Epidemiology, College of Preventive Medicine, Army Medical University (Third Military Medical University), Chongqing, 400038 China

**Keywords:** Lung cancer, Non-coding RNAs

## Abstract

The prognosis of lung cancer is poor with few effective therapies. Targeting ferroptosis is a new promising strategy for cancer therapy. LINC00641 has been involved in several cancers, however, its specific roles in lung cancer treatment remain largely unknown. Here, we reported that LINC00641 was down-regulated in tumor tissues and its downregulation was associated with poor outcomes in lung adenocarcinoma. LINC00641 was localized primarily in the nucleus and was modified by m^6^A. The nuclear m^6^A reader YTHDC1 regulated LINC00641 expression by affecting its stability. We demonstrated that LINC00641 suppressed lung cancer by inhibiting migration and invasion in vitro and metastasis in vivo. Knockdown of LINC00641 upregulated HuR protein level (especially in the cytoplasm), which subsequently increased N-cadherin levels by stabilizing its mRNA, then ultimately promoted EMT. Interestingly, LINC00641 knockdown in lung cancer cells increased the arachidonic acid metabolism and promoted ferroptosis sensitivity. Our findings identified LINC00641 as a tumor suppressor through inhibiting EMT. In another aspect, low expression of LINC00641 caused a ferroptotic vulnerability in lung cancer cells, which may serve as a potential ferroptosis-related therapeutic target for lung cancer.

## Introduction

Lung cancer is the leading cause of cancer-related death, and the incidence rate is further increasing in China and worldwide [1, 2]. Lung adenocarcinoma (LUAD) is the most frequent pathological type of lung cancer [3]. The genetic (such as p53 mutation) and epigenetic (such as histone modification, DNA or RNA methylation, and noncoding RNA expression) alterations are two key drivers in lung tumorigenesis and cancer progression [4]. Great progress has been made in discovering new biomarkers and actionable drug targets based on genetic and epigenetic alterations, however, the overall 5-year survival rate of lung cancer is only about 20% [5]. New therapeutic targets and strategies are urgently needed.

Long noncoding RNAs (lncRNAs) are defined as transcripts >200 nucleotides in length with no protein coding potential [6]. LncRNAs regulate gene expression at pre-transcriptional, transcriptional and post-transcriptional levels by interacting with biomolecules (such as proteins, RNA or DNA) in cells, thereby regulating tumor proliferation, invasion, migration, angiogenesis, immune evasion, and other hallmarks of cancer [7]. We previously screened with Affymetrix GeneChip in primary lung tumor and paired adjacent normal lung tissues and found lncRNA-LINC00641 (NR_038970) significantly downregulated in lung tumor tissues [8]. LINC00641 has been reported to exert various biological functions in several cancers by acting as a competitive endogenous RNA (ceRNA). For example, LINC00641 inhibited cell proliferation, migration, as well as invasion by sponging miR-194-5p in breast cancer [9]. LINC00641 hindered the progression of cervical cancer and bladder cancer through miR-378a-3p/CPEB3 and miR-197-3p/KLF10/PTEN/PI3K/AKT cascade, respectively [10, 11]. LINC00641 was also involved in lung cancer by sponging miR-424-5p to upregulate PLSCR4 [12]. However, the ceRNA mechanism only accounted for the functions of LINC00641 in the cytoplasm. Despite the mainly nuclear localization of LINC00641, its biological mechanism in the nucleus has not yet been uncovered. In addition, its specific roles in lung cancer treatment remain largely unknown.

Epithelial-mesenchymal transition (EMT) drives tumor cells metastasis [13]. During the entire EMT process, the epithelial cell characteristics decrease (such as E-cadherin) and the mesenchymal markers increase (such as N-cadherin and vimentin) [14]. EMT plays a crucial role in promoting tumor initiation and metastasis [15]. EMT is also associated with immunosuppression and resistance to therapies, including chemotherapy and immunotherapy [16, 17]. Uncovering how EMT process is regulated during cancer development is essential for developing effective biomarkers and therapy for lung cancer patients.

Ferroptosis is a newly defined form of programmed cell death that can be executed by phospholipid peroxidation, a process arising from the reaction between iron, reactive oxygen species (ROS) and phospholipids containing polyunsaturated fatty acid chains (PUFA-PLs) [18]. Ferroptosis has been proposed as a novel approach to reverse drug resistance in cancer therapy [19]. The biological processes that modulate ferroptosis-promoting or surveillance molecules, redox and iron homeostasis, and cell metabolism could provide potential cancer therapy by triggering ferroptosis. The sensitivity of ferroptosis is highly dependent on the state of cellular metabolism, especially the metabolism of lipids, iron, and amino acids [20]. In addition, cancer cells undergoing EMT are often resistant to various treatments, but at the same time displaying high susceptibility to ferroptosis [21–23]. For example, E-cadherin-mediated intercellular interaction inhibits EMT but suppresses ferroptosis through intracellular Merlin-Hippo signaling in epithelial cells [24].

Growing evidence indicates that lncRNAs are key regulators of EMT, abnormal lipid metabolism, and ferroptosis. Exploring the roles of lncRNAs in these biological processes may provide new strategies for cancer treatment. In this study, we demonstrated that LINC00641 downregulation promoted EMT process through regulating RNA-binding protein Human antigen R (HuR). Interestingly, low expression of LINC00641 coincidently conveyed a ferroptotic vulnerability in lung cancer.

## Materials and methods

### Cell lines, animals and reagents

The lung cancer cell lines A549, H1299, H1975 and a normal lung epithelial cell (HBE) were obtained from the Cell Bank of the Chinese Academy of Science (Shanghai, China) or the American Type Culture Collection (ATCC, Manassas, VA, USA), cultured in RPMI-1640 (Gibco, Life Technology, Carlsbad, CA, USA) supplemented with 10% fetal bovine serum (Gibco). All the cells were maintained at 37 °C with 5% CO_2_. The male BALB/c-nude mice were purchased from Vital River Laboratory Animal Technology Co., Ltd. (Beijing, China). The (1S, 3R)-RSL3, Erastin, Arachidonic acid (AA), IFN-γ, and Actinomycin D were purchased from MCE (New Jersey, USA).

### RNA extraction, cDNA synthesis and qRT-PCR analysis

The total RNA from cells were extracted by using Eastep Super Total RNA Extraction Kit (Promega, Shanghai, China). The nuclear and cytoplasmic RNA were extracted by using PARIS™ Kit (Invitrogen, NY, USA). After cDNA synthesis using PrimeScript™ RT reagent Kit with gDNA Eraser (TaKaRa, Dalian, China), the real-time PCR was conducted using the SYBR Premix Ex Taq (TaKaRa). *β-actin* were used to calculate the relative gene expression levels using 2^^−(ΔΔCt)^ method. Primers sequences were provided in Supplementary Table S1.

### Rapid amplification of cDNA ends

Rapid amplification of cDNA ends (RACE) was performed following the protocol of the RACE System. Briefly, first-strand cDNA was synthesized using a gene-specific primer. The original mRNA template was removed by RNase H and the cDNA was purified followed by tailing with dCTP using TdT. A nested PCR was then carried out to amplify cDNA. Primers sequences are provided in Supplementary Table S1.

### Plasmid construction and cell transfection

For LINC00641 and YTHDC1 knockdown, shRNA and corresponding control lentiviruses were synthesized by GeneChem (Shanghai, China). The infected cells were selected using puromycin (2 μg/ml; Biosharp, Anhui, China) to construct stable knockdown cells. For knockdown of HuR, siRNAs targeting HuR were purchased from the GenePharma (Shanghai, China). The siRNAs were transfected into cells using Lipofectamine 2000 (Invitrogen). LINC00641 shRNA, YTHDC1 shRNA and HuR siRNA target sequences were shown in Supplementary Table S1.

### Western blot

Western blot (WB) was performed as described previously [8]. The antibodies used in this study were rabbit polyclonal to YTHDC1 (1:1000; Abcam Inc., Cambridge, MA), rabbit monoclonal to HuR (1:1000; Abcam), rabbit monoclonal to GAPDH (1:1000; Abcam), rabbit monoclonal to GPX4 (1:1000; Abcam), rabbit monoclonal to vimentin (1:1000; Abcam), rabbit polyclonal to histone H3 (1:1000; ABclonal, Wuhan, China), rabbit monoclonal to N-cadherin (1:1000; ABclonal), rabbit polyclonal to E-cadherin (1:1000; ABclonal), and rabbit monoclonal to SLC7A11 (1:1000; ABclonal).

### RNA immunoprecipitation assay and N6-methyladenosine immunoprecipitation assay

RNA immunoprecipitation (RIP) assay in lung cancer cells was performed using EZ-Magna RIP^TM^ RNA-Binding Protein Immunoprecipitation Kit (Millipore, Billerica, MA, USA). Briefly, cells were lysed using the RIP lysis buffer. Immunoprecipitation were conducted with magnetic beads linked with anti-HuR, anti-YTHDC1 or anti-IgG. The enrichment of LINC00641 and *CDH2* mRNA was assessed by qRT-PCR assay. The MeRIP was performed to determine the N6-methyladenosine (m^6^A) levels of LINC00641. Briefly, 5 μg anti-m^6^A antibody (ABclonal) or normal rabbit IgG were bound to magnetic beads (Millipore). A total of 50 μg RNA from the cells was immunoprecipitated in RIP buffer (Millipore) overnight at 4 °C. After treating with proteinase K (10 mg/mL), RNAs were then extracted with an RNeasy Mini Kit (Qiagen). The enrichment of LINC00641 in m^6^A immunoprecipitated RNAs was assessed by qRT-PCR assay. Primers sequences were provided in Supplementary Table S1.

### RNA stability detection

Lung cancer cells (3.0 × 10^5^) were seeded into 6-well plates and were then treated with Actinomycin D (5 μg/mL). Cells were harvested for RNA purification. LINC00641 and *CDH2* mRNA expression levels were subsequently detected by qRT-PCR to test the stability of LINC00641 or *CDH2* mRNA.

### Cell viability assay in vitro

The cell viability was detected using Cell Counting Kit-8 (CCK-8) assay. Cells were seeded into 96-well plate (3 × 10^3^ cells/well) and cell viability were assessed using the CCK-8 (Dojindo Laboratories, Kumamoto, Japan) on days 1, 2, 3, 4 and 5, or under different treatment of reagents (RSL3, AA or IFN-γ). All of the experiments were performed in triplicate.

### Colony formation assays

Cells were seeded in 6-well plates overnight and grown for 9 days. Media containing 10% FBS were changed every 3 days and colonies were fixed with methanol for 30 min and then stained with 0.1% crystal violet (Beyotime Biotechnology, Shanghai, China) for 15 min. The visible colonies were then counted. All of the experiments were performed in triplicate.

### Cell migration and invasion assays

For the migration assays, cells in serum-free media were seeded into the upper chamber of the insert (Corning, New York, USA). For the invasion assays, cells in serum-free medium were seeded into the upper chamber of the insert coated with Matrigel (Corning). Medium containing 10% fetal bovine serum was added to the lower chamber. After incubation, the cells remaining on the upper membrane were removed with cotton wool. The migrated or invaded cells were stained with 0.1% crystal violet. All experiments were conducted in triplicate.

### Animal experiments in vivo

For subcutaneous xenograft model, eight male BALB/c-nude mice weighing 18–23 g were randomly divided into two groups equally. A549 cells (5 × 10^6^) with LINC00641 stable knockdown or control were suspended in 200 μL of 50% Matrigel (Corning), then injected subcutaneously into the flanks of the nude mice. For the metastatic tumor model, the male BALB/c-nude mice weighing 18–23 g were randomly divided into two groups. A549 cells with LINC00641 stable knockdown or control were injected into the tail veins of the nude mice (2 × 10^6^ cells per mice). The imaging of cancer metastasis was monitored with micro-CT at 7 weeks post injection. The mice were sacrificed 7 weeks after injection and the lungs were removed for further analysis. All experimental animal procedures were approved by the Experimental Animal Welfare Ethics Committee, Zhongnan Hospital of Wuhan University.

### Micro-computed tomography (micro-CT) scanning

The mice were anesthetized with 1% pentobarbital sodium (100 ml/kg body weight) prior to the scanning. Micro-CT was performed with the following parameters: 20.3 μm, 1 degree at an angle of increment and 360 views. Each mouse was scanned and the images of the chest were reconstructed.

### Transcriptome sequencing and analysis

The total RNAs were extracted from A549 cells and the ribosomal RNAs (rRNA) were removed by using ribosomal RNA removal kit. After cDNA synthesis with DNA Polymerase I and RNase H, the cDNA products were enriched using PCR and were selected according to the fragment size to create the cDNA library. The cDNA library was sequenced using a next-generation sequencing based on Illumina HiSeq System. Then sequencing reads filtering genome mapping, gene expression analysis and differentially expressed genes (DEG) detection were conducted based on the raw data.

### Immunofluorescence cell staining

A549 cells were seeded on sterile confocal petri dish (Biosharp), fixed in methyl alcohol for 15 min, permeabilized by 0.1% Triton X-100, and then blocked with 1% BSA for 30 min. The cells were then incubated with rabbit monoclonal to HuR (Abcam) overnight at 4 °C. After washing three times with PBST, the cells were probed with Alexa-Fluor-555-conjugated goat anti-rabbit-IgG (Abcam) for 1 h at room temperature, followed by nuclear counterstaining with DAPI (Beyotime). Coverslips were observed with a fluorescence microscope (ZEISS, Germany).

### N6-methyladenosine dot blot

Total RNA were denatured at 65 °C for 5 min in RNA incubation buffer, and then spotted on two sets of the positively charged nylon membrane (Beyotime). After UV crosslinking, the membrane was washed with 1×TBST buffer. One of the membranes was dyed directly with the methylene blue staining, and the other was blocked with 5% non-fat milk and incubated with anti-m^6^A antibody (1:1000, ABclonal) overnight at 4 °C. Then HRP-conjugated goat anti-rabbit IgG (Beyotime) was incubated for 1 h at room temperature. The membrane was then detected with ECL Prime Western Blotting Detection Reagent.

### Cellular ROS measurement

A549 cells (2 × 10^5^) were treated with RSL3 (1 μmol/L) for 24 h, followed by the addition of DHE (KeyGEN BioTECH, Jiangsu, China) for 60 min. Cells were washed with PBS and harvested with trypsin/EDTA (0.25%; Gibco), followed by washing twice with PBS. Cells were subjected to flow cytometry to measure the levels of cellular ROS.

### Iron assays

The total iron and the intracellular ferrous levels were determined using the iron assay kit (Abcam) following the manufacturer’s instructions. Briefly, cells were centrifuged at 4 °C (13,000 × g, 10 min), and then homogenized with 2× volume iron tube buffer solution. To measure total Fe, 5 µl of Iron Reducer was added to each sample. To measure ferrous iron, 5 µl of iron assay buffer was added to each sample. The reactions were incubated for 30 min at room temperature in dark conditions. Then, each sample was added with 100 µl of iron probe and incubated in a horizontal shaker without light for 60 min. Finally, the absorbance was measured at 593 nm.

### Luciferase reporter assay

To verify the effect of m^6^A modification on LINC00641, we mutated the A to G at the 3 predicted potential m^6^A binding sites. The wild type (A) or mutant type (G) sequences of LINC00641 were inserted into the pmirGLO luciferase vectors. A549 cells (5 × 10^3^) were seeded into 96-well plates in triplicate for each group. The pmirGLO-LINC00641-wild type (wt) or pmirGLO-LINC00641-mutant (mut) was transfected in A549 cells. Firefly and Renilla luciferase activities were measured at 48 h after transfection using the Dual-Luciferase Assay System (Beyotime) following the manufacturer’s instructions.

### Flow cytometry and cell sorting

A549 cells (5 × 10^6^) were suspend in cell staining buffer (BioLegend, California, USA). Firstly, non-specific binding was blocked using Human TruStain FcX™ (5 µl per million cells in 100 µl staining volume, BioLegend) for 15 min. Then the FITC anti-human CD324 (E-Cadherin) antibody (BioLegend) and APC anti-human CD325 (N-Cadherin) antibody (BioLegend) were incubated on ice for 20 min in the dark. After washing with Cell Staining Buffer, flow cytometry was performed to select the target cells. Isotype controls were included in all flow cytometry experiments.

### RNA pulldown

Biotin-labeled LINC00641 was obtained through in vitro transcription from vector pcDNA3.1 using Biotin RNA Labeling Mix (Roche, Mannheim, Germany) and T7 RNA polymerase (Roche). After treating with RNase-free DNase I (Promega, Madison, WI, USA), the biotin-labeled RNAs were purified with the RNeasy Mini Kit (Qiagen) and then added into the A549 cell lysates. After incubating at 4 °C for 2 h, the biotin-labeled RNAs were immunoprecipitated with Dynabeads^TM^ M-270 Streptavidin (Invitrogen). Proteins were then eluted from the dynabeads and detected by WB.

### Statistical analysis

Statistical analyses were performed using the SPSS 25.0 software (SPSS, Inc., Chicago, IL, USA). Differences between two groups were analyzed with the two-tailed Student’s *t*-test or Mann–Whitney *U*-test. The one-way analysis of variance (ANOVA) with Tukey’s multiple comparison test was used to assess differences between more than two groups. The differences between cell proliferation curves were evaluated using repeated measures analysis of variance. A two-sided *P*-value less than 0.05 was taken as statistically significant.

## Results

### LINC00641 is down-regulated and its decreased expression is associated with progression and poor prognosis in LUAD

**Fig. 1:**
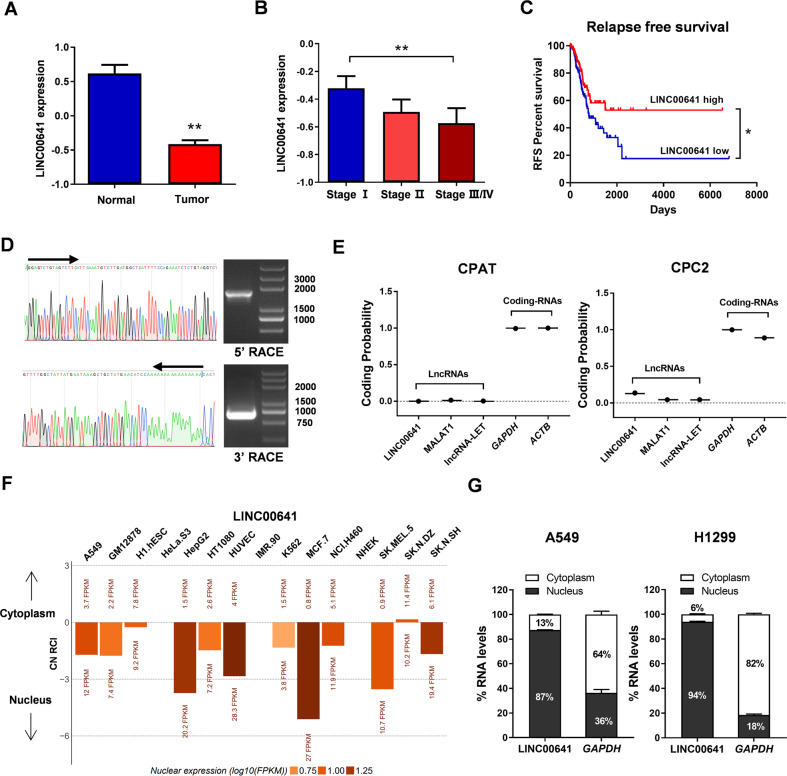
LINC00641 expression is down‐regulated in LUAD and its characterization. **A** LINC00641 expression data in TCGA LUAD dataset (58 normal vs 488 tumor tissues) was downloaded from the TANRIC website (https://ibl.mdanderson.org/tanric/_design/basic/main.html). The results were expressed as the mean ± SEM. **Mann–Whitney *U*‐test, *P* < 0.01. **B** LINC00641 expression in tumor tissues of different TNM stages in TCGA LUAD dataset. The results were expressed as the mean ± SEM. **Kruskal Wallis test, *P* < 0.01. **C** Kaplan–Meier curves for relapse free survival (RFS) of LUAD patients expressing high and low expression levels of LINC00641 in TCGA LUAD dataset. *Log‐rank test, *P* < 0.05. **D** Rapid amplification of cDNA ends (RACE) assays in A549 cells to detect the whole sequence of LINC00641. Left: sequencing of PCR products indicated the boundary between the universal anchor primer and LINC00641 sequences. Right: a gel electrophoresis image of PCR products from the 5′-RACE and 3′-RACE assays. **E** The coding potential of LINC00641. Coding potentials of lncRNA (LINC00641, MALAT1 and lncRNA-LET) and mRNA (*GAPDH*, *ACTB*) were calculated using CPAT (https://wlcb.oit.uci.edu/cpat/) and CPC2 (http://cpc2.gao-lab.org/index.php). **F** The subcellular localization of LINC00641 in LncATLAS database. **G** qRT-PCR detection of the percentage of LINC00641 and *GAPDH* in the nuclear and cytoplasmic fractions of A549 and H1299 cells. *GAPDH* served as the cytoplasmic localization control.

We first explored the expression pattern of LINC00641 in LUAD using TCGA database, which showed LINC00641 was significantly down-regulated compared to the normal tissues (Fig. 1A). The expression levels of LINC00641 were also significantly decreased in patients with advanced TNM stage (Fig. 1B). LINC00641 expression levels were also assessed in a normal lung epithelial cell line (HBE) and three lung cancer cell lines (H1299, H1975 and A549). The results showed that the LINC00641 expression in lung cancer cell lines was lower than that in HBE cell line (Supplementary Fig. S1A). Kaplan–Meier survival analysis was performed to assess the clinical significance of LINC00641 in LUAD. The results showed that low expression of LINC00641 was significantly associated with poor relapse free survival (RFS) (*P* < 0.05; Fig. 1C). The association with RFS in LUAD patients remained significant in the multivariate Cox proportional hazards regression analysis after adjustment for age, gender, smoking and tumor stage (Hazard ratio = 0.642, 95% confidence interval = 0.421–0.980, *P* = 0.040). Collectively, these results revealed that LINC00641 was down‐regulated in LUAD and its low expression level was associated with poor prognosis in LUAD.

### LINC00641 is a long non-coding RNA and is mainly located in the nucleus

LINC00641 was located at chromosome 14q11.2. The RACE assays identified the full length of LINC00641 with 3185nt (Fig. 1D and Supplementary Fig. S1B). We next investigated whether LINC00641 was a noncoding gene. LINC00641 harbored 50 potential open reading frames (ORF) that might code short peptides of 11–103 amino acids predicted by the ORF Finder (https://www.ncbi.nlm.nih.gov/orffinder/; Supplementary Fig. S1C). All the ORFs lacked strong Kozak consensus sequence, suggesting LINC00641 as a non-coding RNA. The non-coding potential of LINC00641 was further confirmed by coding potential assessment tool (CPAT; https://wlcb.oit.uci.edu/cpat/) and coding potential calculator (CPC2; http://cpc2.gao-lab.org/index.php; Fig. 1E). We next detected the subcellular localization of LINC00641. By using the LncATLAS, we found that LINC00641 was expressed in both cytoplasm and nucleus, and was mainly located in the nucleus in most cell lines (including A549 cell line) (Fig. 1F). We further separated the nuclear and cytoplasmic fractions and confirmed that LINC00641 was mainly located in the nuclear fractions of A549 and H1299 cells (Fig. 1G).

### LINC00641 is modified by m^6^A and its stability is maintained by nuclear m^6^A reader YTHDC1

**Fig. 2:**
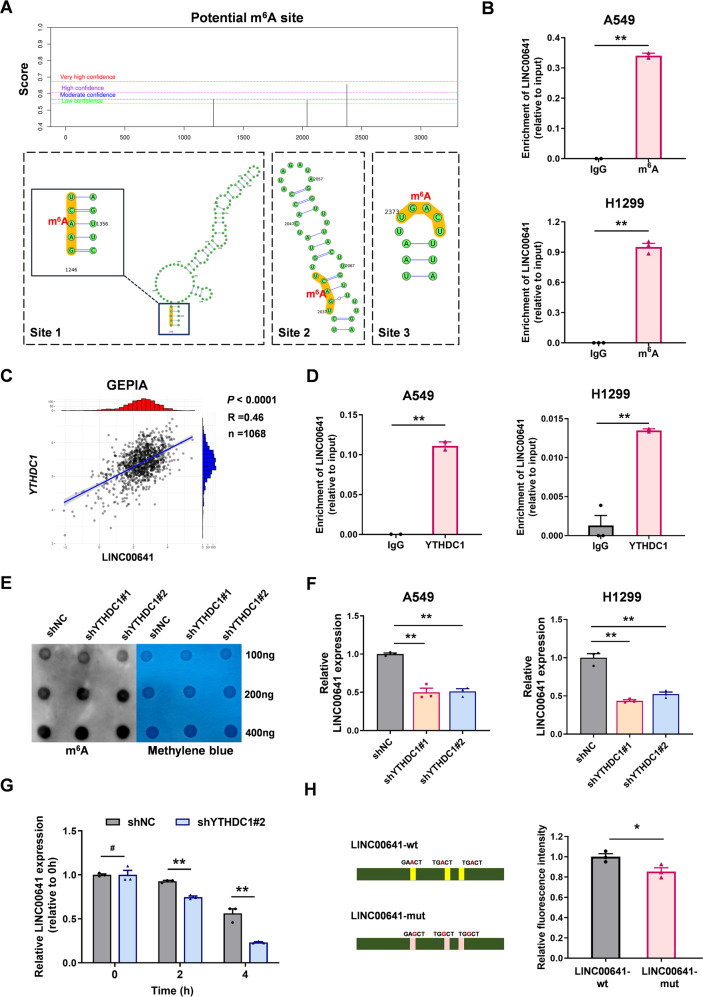
Nuclear m^6^A reader protein YTHDC1 binds to LINC00641 and affects its stability. **A** The m^6^A sites in LINC00641 sequence and the corresponding secondary structures was predicted using SRAMP prediction server (http://www.cuilab.cn/sramp). **B** The m^6^A RNA modification of LINC00641 was detected by MeRIP assays in A549 and H1299 cells. **C** The correction between LINC00641 and *YTHDC1* mRNA in GEPIA lung cancer database. **D** The binding effects of YTHDC1 to LINC00641 was detected by RIP assay in A549 and H1299 cells. **E** The overall m^6^A modification level was detected by dot blot in A549 cells with stable knockdown of YTHDC1. **F** The expression levels of LINC00641 were detected by qRT-PCR in A549 and H1299 cells after knockdown of YTHDC1. **G** Stability of LINC00641 was measured by qRT-PCR relative to time 0 h after blocking new RNA synthesis with Actinomycin D (5 μg/mL) in A549 cells with stable knockdown of YTHDC1. **H** The effect of m^6^A modification on the stability of LINC00641 was detected by dual-luciferase assay in A549 cells. Three DRACH motif (D = G/A/U, R = G/A, H = A/U/C) were replaced with DRGCH and inserted the sequences into the pmirGLO vectors to generate pmirGLO-LINC00641-wt and pmirGLO-LINC00641-mut vectors. #*P* > 0.05, *P < 0.05, **P < 0.01.

We then probed the potential mechanisms that controlled the tissue-specific expression of LINC00641 in lung cancer. Previous studies have demonstrated that m^6^A modification was the most extensively epigenetic modification at RNA in the nucleus [25]. By using SRAMP prediction server (http://www.cuilab.cn/sramp) [26], a mammalian m^6^A sites predictor, we identified three m^6^A sites in LINC00641 sequence (Fig. 2A). We then conducted the methylated RNA immunoprecipitation (MeRIP)-qPCR assays and confirmed that LINC00641 was modified by m^6^A in A549 and H1299 cells (Fig. 2B). Among various kinds of m^6^A readers, YTHDC1, to our knowledge, was reported as the only YTH domain-containing nuclear reader, which played multiple roles in gene expression, including RNA splicing, RNA export, and RNA decay [27]. Given the nuclear localization of LINC00641, we speculated that LINC00641 could be recognized and regulated by m^6^A nuclear reader YTHDC1. To confirm this hypothesis, we first found a significant correlation between YTHDC1 and LINC00641 expression in lung cancer by using GEPIA database (Fig. 2C). RIP-qPCR assays revealed that the YTHDC1 could directly bind to LINC00641 (Fig. 2D).

To clarify whether LINC00641 was regulated by YTHDC1, we established loss-of-function cell models by transfecting two independent shRNAs targeting YTHDC1 into the A549 and H1299 cells (Supplementary Fig. S2A, B). Knockdown of YTHDC1 did not alter the overall m^6^A modification levels (Fig. 2E). However, knockdown of YTHDC1 resulted in a significant decrease of LINC00641 expression (Fig. 2F). YTHDC1 has been reported to affect gene expression by regulating the stability of m^6^A modified RNA. To further test whether the stability of LINC00641 was affected by YTHDC1, Actinomycin D (ActD) was used to block new RNA synthesis in A549 and H1299 cells. Knockdown of YTHDC1 significantly increased LINC00641 degradation (Fig. 2G and Supplementary Fig. S2C). These findings indicated that m^6^A reader YTHDC1 regulated LINC00641 expression by maintaining its stability. We next designed a dual-luciferase assay to verify the effect of m^6^A modification on the stability of LINC00641. We mutated the three potential m^6^A binding sites by replacing DRACH motif (D = G/A/U, R = G/A, H = A/U/C) with DRGCH and inserted the sequences into the pmirGLO vectors to generate pmirGLO-LINC00641-wt and pmirGLO-LINC00641-mut vectors, respectively (Fig. 2H). We found a significant reduction in luciferase activity in the mutant luciferase reporter, indicating that the stability of LINC00641 was dependent on m^6^A modification (Fig. 2H). Taken together, these findings suggested that YTHDC1 may bind to the three DRACH motif in the LINC00641 and regulate the stability of LINC00641.

**Fig. 3:**
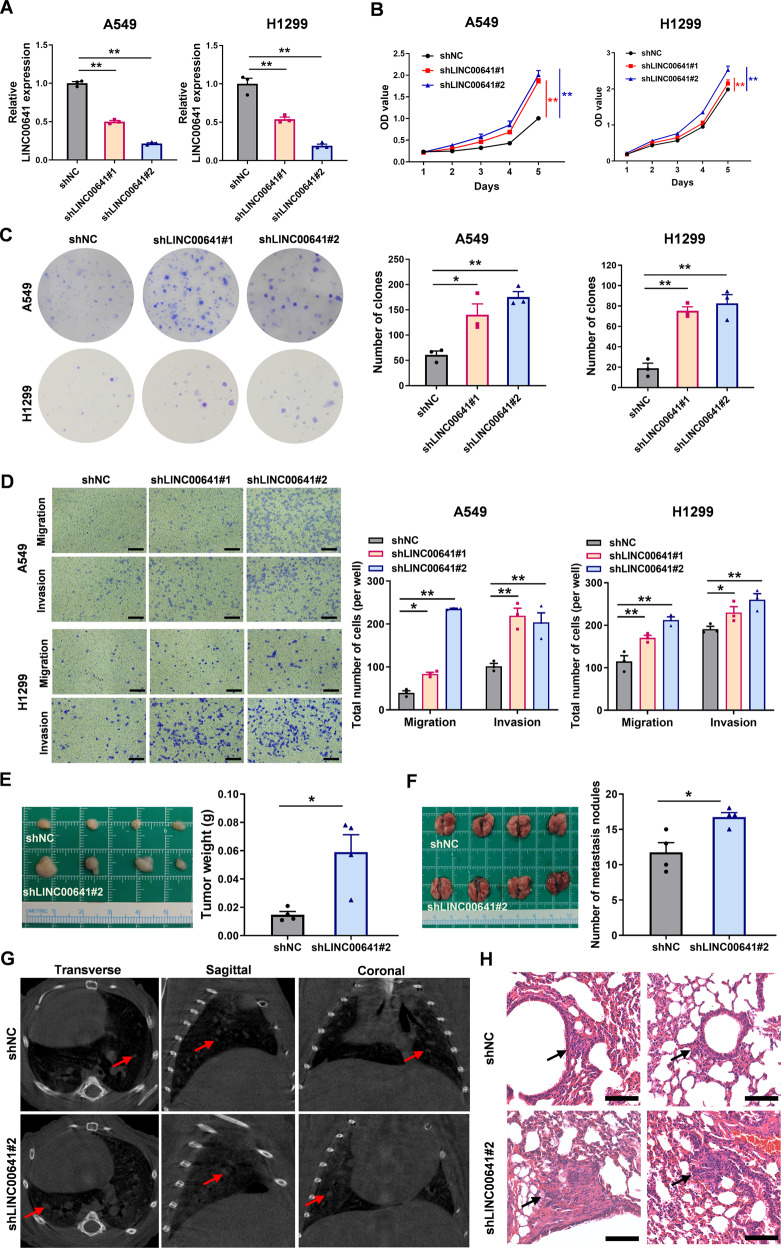
Knockdown of LINC00641 promotes cell proliferation, migration and invasion in vitro and tumor growth and metastasis in vivo. **A** The knockdown effects of LINC00641 by shRNAs were detected using qRT-PCR in A549 and H1299 cells. **B** Cell proliferations were detected by CCK-8 assays after stable knockdown of LINC00641 in A549 and H1299 cells. The difference between cell proliferation curves was evaluated using repeated measures analysis of variance. **C** Colony formation assays were conducted to clarify the effect of LINC00641 knocked down in A549 and H1299 cells. **D** Transwell assays were used to examine the effect of LINC00641 knockdown on migration and invasion in A549 and H1299 cells (scale bar: 200 μm). **E** Left: Subcutaneous xenograft tumors were dissected and photographed. Right: The tumor weight of A549 cells with stable knockdown of LINC00641 or control. **F** Photograph of entire lungs from nude mice in each group 7 weeks after injections of A549 cells with LINC00641 stable knockdown or control. **G** Representative metastatic nodules by micro-CT scanning in the transverse, coronal, and sagittal sections, which were indicated by arrows. **H** Representative H&E staining of lung tissue slices confirmed that more metastatic nodules were present in LINC00641-knockdown group than vector control group (scale bar: 100 μm). *P < 0.05; **P < 0.01.

To determine if a feedback loop existed between LINC00641 and YTHDC1, we used two independent shRNAs to stably knock down LINC00641 in A549 and H1299 cells (Fig. 3A). Our results showed that knockdown of LINC00641 had no significant impact on the expression levels of YTHDC1 (Supplementary Fig. S2D, E).

### LINC00641 knockdown enhances cell proliferation, migration, and invasion in vitro, as well as tumor growth and metastasis in vivo

We next investigated the biological functions of LINC00641 in lung cancer. CCK8 and colony formation assays showed that cell vitality and proliferation were significantly enhanced after LINC00641 knockdown (Fig. 3B, C). Knockdown of LINC00641 also significantly increased the migration and invasion of A549 and H1299 cells compared with vector control (Fig. 3D).

We next tested whether LINC00641 inhibited tumor growth and metastasis in vivo. In the subcutaneous xenograft models, LINC00641 knockdown significantly increased the growth of A549 xenograft tumors in vivo (Fig. 3E). In the metastasis models, the number of metastatic nodules on the lung surface was significantly increased in LINC00641 knockdown group compared to the control seven weeks after injection (Fig. 3F). The lung metastases in mice were also monitored using micro-CT, which showed that lung metastasis displayed highly diffuse tumor morphology, spread throughout the lung in LINC00641 knockdown group compared to the control (Fig. 3G). The representative metastatic nodules were observed in the transverse, coronal, and sagittal sections, and metastatic nodules were found to be larger in LINC00641 knockdown group (Fig. 3G). The differences were further confirmed by the examination of the lungs by hematoxylin and eosin (H&E) staining of the mice lung sections (Fig. 3H). Collectively, our in vivo and in vitro data supported LINC00641 as a tumor suppressor in lung cancer.

### LINC00641 binds to HuR and inhibits the accumulation of HuR in cytoplasm

**Fig. 4:**
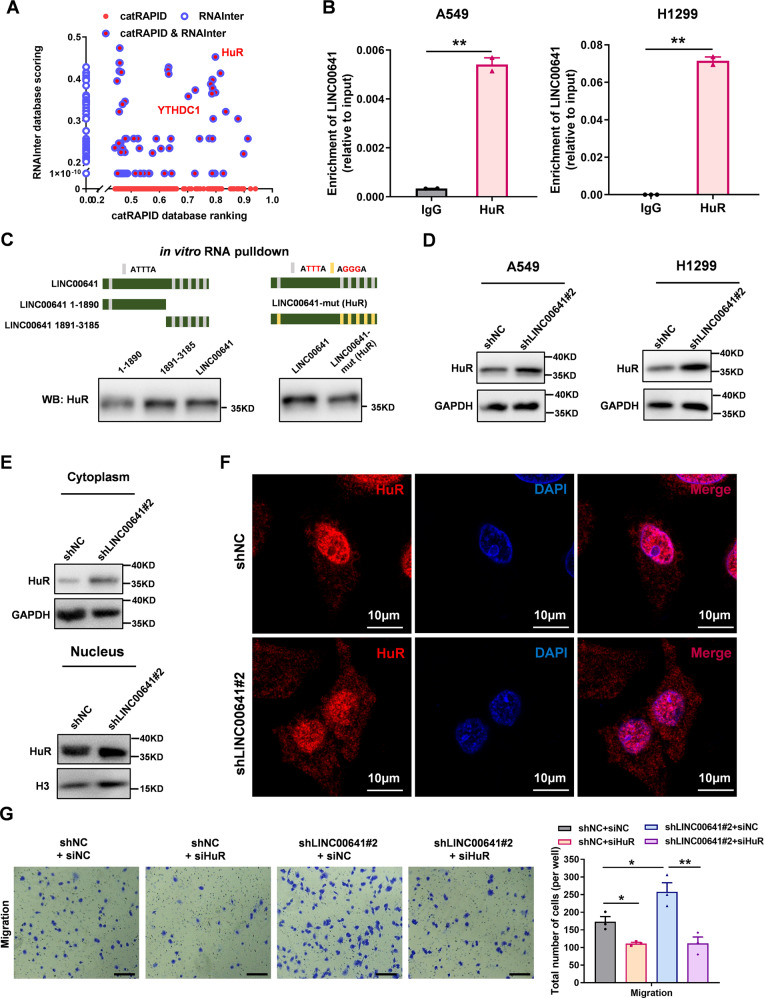
Nuclear LINC00641 binds to HuR and inhibits the accumulation of HuR in cytoplasm. **A** The LINC00641-binding protein was predicted by two RNA-binding protein databases (catRAPID and RNAinter). The graph combined the rankings and scorings of these proteins in the two databases (the lack of hits in either database was denoted as “0”). **B** The binding effects of HuR to LINC00641 was detected by RIP assay in A549 and H1299 cells. **C** Left: The binding region of LINC00641 by HuR protein was detected by RNA pulldown assays, which display that HuR mainly bind to the 1891–3185nt fragment of LINC00641. Right: The binding effects of HuR with wild type or mutant LINC00641 were detected by RNA pulldown assays. HuR showed stronger binding affinity with wild-type LINC00641 than with mutant type LINC00641. **D** The protein levels of HuR were detected by WB in A549 and H1299 cells after knockdown of LINC00641. **E** The cytoplasmic and nuclear protein levels of HuR were detected by WB in A549 cells after knockdown of LINC00641. **F** The localization of HuR in cytoplasm and nucleus were detected using a confocal microscope in A549 cells with LINC00641 stable knockdown or control. **G** Rescue effect of HuR siRNAs on migration in A549 cells with LINC00641 stable knockdown or control (scale bar: 200 μm). *P < 0.05, **P < 0.01.

LncRNAs typically rely on RNA-binding proteins (RBPs) to perform their biological functions, as they often interact with RBPs to form regulatory complexes that can modulate gene expression and other cellular processes. To explore the possible regulatory mechanism of LINC00641, we systematically evaluated LINC00641’s binding potentials to proteins by integrative analysis with two RNA binding protein databases: catRAPID (http://service.tartaglialab.com/page/catrapid_omics2_group) and RNAinter (http://www.rnainter.org/) [28], which identified 2065 and 310 LINC00641-protein interactions, respectively. Combining the rankings and scorings of these proteins in the two databases (the lack of hits in either databases was denoted as “0”), we found that HuR (an RNA stabilizer protein encoded by *ELAVL1* gene [29]) showed the highest overall ranking (Fig. 4A). Our subsequent RIP-qPCR experiments confirmed that HuR could bind to LINC00641 in A549 and H1299 cells (Fig. 4B). We further verified the binding regions of HuR to the LINC00641 using in vitro RNA pulldown assays. We found six potential binding sites of HuR (ATTTA) on LINC00641, and five of them were located within the region from 1891 to 3185nt. Therefore, we divided LINC00641 into two fragments: fragment 1–1890nt and fragment 1891–3185nt. RNA pulldown assays showed that HuR mainly bound to the 1891–3185nt fragment of LINC00641 (Fig. 4C, left). We next introduced mutations on the 6 ATTTA sites of LINC00641 to AGGGA and observed a reduction in the binding ability of the mutant LINC00641 to HuR protein (Fig. 4C, right), demonstrating that the ATTTA on LINC00641 might be the binding sites of HuR.

We then examined the mutual regulation of LINC00641 and HuR protein. Knockdown of HuR protein using siRNAs did not altered the expression levels of LINC00641 (Supplementary Fig. S3A), ruling out the regulatory effect of HuR on LINC00641. However, LINC00641 knockdown upregulated the HuR protein levels in A549 and H1299 cells (Fig. 4D). HuR protein was distributed in both cytoplasm and nucleus [30]. It has been reported that the carcinogenic effect of HuR protein was mainly achieved by the cytoplasmic HuR [31]. We next sought to analyze the cytoplasmic and nuclear protein levels of HuR by separating the cytoplasm and nucleus. We found that HuR protein levels were markedly upregulated in the cytoplasm after knockdown LINC00641 (Fig. 4E). The accumulation of HuR in cytoplasm was confirmed by using confocal microscopy (Fig. 4F). The regulating effect of LINC00641 on HuR suggested that HuR might mediate the tumor suppressor function of LINC00641 in lung cancer cells. To prove this, we transfected siRNAs targeting *HuR* into A549 cells with LINC00641 stable knockdown or control cells (Supplementary Fig. S3B). We found that the knockdown of HuR abrogated the effect of LINC00641 on the cell migration (Fig. 4G**)**. Taken together, these results demonstrated that LINC00641 acted as a tumor suppressor in lung cancer partially through HuR.

### LINC00641 regulates EMT process and CDH2 is a direct target gene of LINC00641-HuR axis

**Fig. 5:**
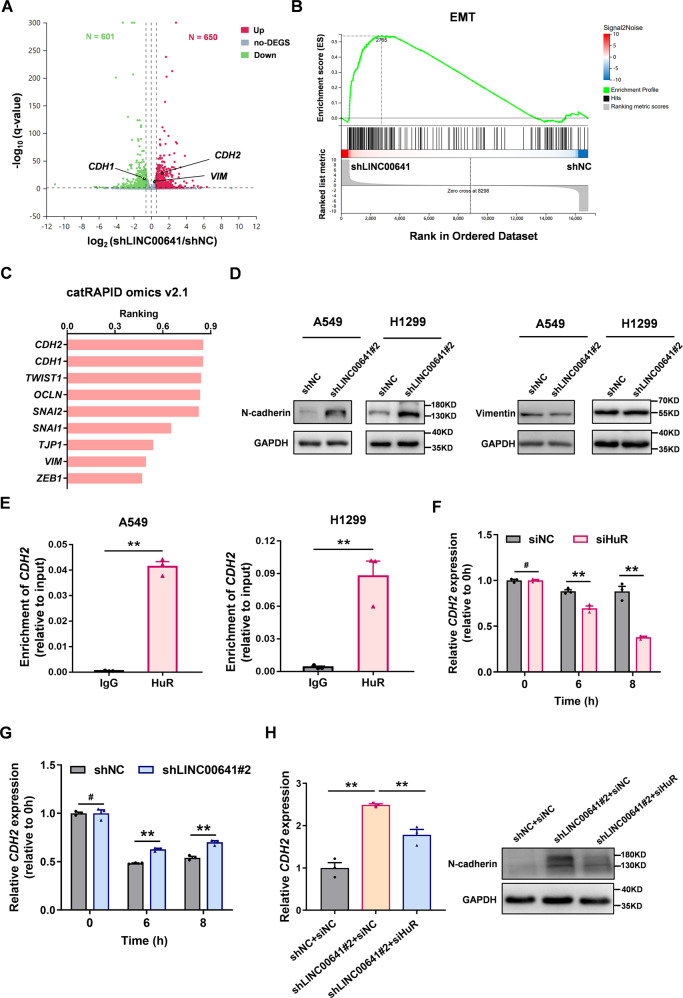
LINC00641 knockdown promotes N-cadherin by enhancing the stability of *CDH2* mRNA through HuR. **A** The volcano plot showed the differentially expressed genes induced by stably knockdown of LINC00641 in A549 cells. Green dots indicated downregulated genes; red dots indicated upregulated genes. **B** Gene set enrichment analysis (GSEA) showed that EMT was altered in LINC00641 knockdown cells compared with control cells in A549 cells. **C** The binding effect of HuR protein and EMT related genes was predicted by using catRAPID omics v2.1. **D** The protein levels of N-cadherin and vimentin were detected by WB in A549 and H1299 cells after knockdown of LINC00641. **E** The binding effects of HuR to *CDH2* mRNA was detected by RIP assay in A549 and H1299 cells. **F** Stability of *CDH2* mRNA was measured by qRT-PCR relative to time 0 h after blocking new RNA synthesis with Actinomycin D (5 μg/mL) in A549 cells with knockdown of HuR using siRNA. **G** Stability of *CDH2* mRNA was measured by qRT-PCR relative to time 0 h after blocking new RNA synthesis with Actinomycin D (5 μg/mL) in A549 cells with stable knockdown of LINC00641. **H** Rescue effect of HuR siRNA on the expression levels of *CDH2* mRNA and N-cadherin in A549 cells with LINC00641 stable knockdown or control. #*P* > 0.05; **P < 0.01.

We next wanted to identify the molecular pathways by which LINC00641 suppressed lung cancer. We performed an RNA-sequencing analysis on A549 cells with stable knockdown of LINC00641, comparing them to control cells. Our analysis revealed 1251 differentially expressed genes (DEGs), with 650 genes upregulated and 601 genes downregulated (*q*-value < 0.05, fold change >1.5) (Fig. 5A). The top 30 upregulated and downregulated genes were also presented by the heatmap in Supplementary Fig. S4A. Gene Ontology (GO) analysis showed that the dysregulated genes were significantly enriched in cell adhesion and cell migration (Supplementary Fig. S4B). The GSEA (Gene set enrichment analysis) showed that the upregulated genes in LINC00641 knockdown cells were positively correlated with EMT, suggesting EMT might be activated after LINC00641 knockdown (Fig. 5B). EMT has been well known to be associated with tumor initiation and metastasis [32]. The promoting effect of LINC00641 knockdown on EMT indicated that LINC00641 may function as a tumor suppressor by regulating EMT.

We next tried to find the LINC00641-HuR axis target genes that were involved in EMT process. HuR was well-known to pervasively bind numerous target RNAs to enhance their stability [33]. We firstly explored the binding effect of HuR to EMT related genes, including EMT transcription factors (*ZEB1/TWIST1/SNAI1/SNAI2*), mesenchymal markers (*CDH2/VIM*), and epithelial markers (*CDH1/OCLN/TJP1*), and found that *CDH2* mRNA showed the highest ranking of binding affinity with HuR (Fig. 5C). We next detected the expressions of these EMT related genes using qRT-PCR and confirmed that knockdown of LINC00641 could promote the mRNA expression levels of *CDH2* and *VIM* in both A549 and H1299 cells **(**Supplementary Fig. S4C). We then validated the protein levels of N-cadherin and vimentin, encoded by CDH2 and VIM, respectively. In LINC00641 knockdown A549 and H1299 cells, WB assays showed an upregulation of N-cadherin, but not vimentin (Fig. 5D). These results suggested that HuR mediated the regulating effect of LINC00641 on CDH2. To further verify this, the RIP-qPCR assays showed that HuR protein could bind to *CDH2* mRNA in A549 and H1299 cells (Fig. 5E). To test whether the stability of *CDH2* mRNA was affected by HuR and LINC00641, ActD treatment were conducted. The qRT-PCR assays showed that knockdown of HuR promoted the *CDH2* mRNA degradation (Fig. 5F), while LINC00641 knockdown promoted the *CDH2* mRNA stability (Fig. 5G) in A549 cells. Moreover, knockdown of HuR abrogated the promoting effect of LINC00641 knockdown on *CDH2* mRNA and N-cadherin protein (Fig. 5H**)**. These results demonstrated that CDH2 is a direct target gene of LINC00641-HuR axis.

### Low expression of LINC00641 provides a ferroptotic vulnerability for lung cancer treatment

**Fig. 6:**
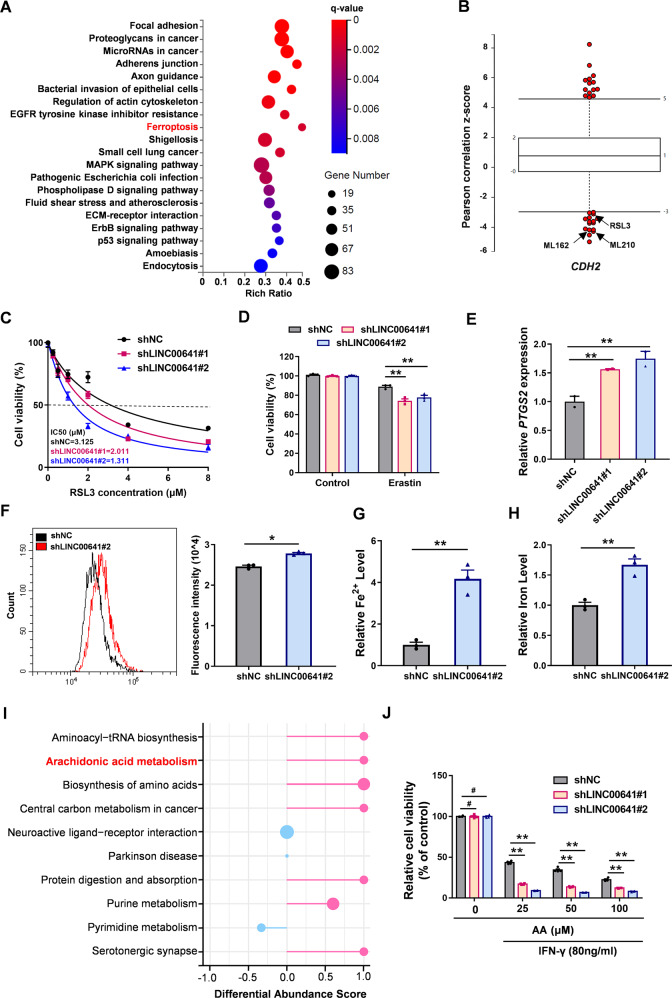
Low expression of LINC00641 provides a ferroptotic vulnerability for lung cancer treatment. **A** Kyoto encyclopedia of genes and genomes (KEGG) pathway analysis were conducted using differentially expressed genes. **B** The expression of *CDH2* was negatively correlated with the resistance to ferroptosis inducer, including ML162, ML210 and RSL3, in the Cancer Therapeutics Response Portal. **C** RSL3 (a ferroptosis inducer) treatment was conducted to clarify the effect of LINC00641 on the ferroptosis in A549 cells for 3 days. Cell viability was detected by CCK-8 assays and IC50 curves were generated using Graphpad Prism 8.0. **D** CCK-8 assays were conducted to clarify the effect of LINC00641 on the Erastin (1 μM)-induced ferroptosis in A549 cells. **E** The expression levels of *PTGS2* mRNA were detected in A549 after knockdown of LINC00641. **F** ROS were detected by flow cytometry. A549 cells were treated with RSL3 (1 μM) for 24 h, followed by incubation with DHE for 60 min at 37 °C. **G**, **H** Level of ferrous iron and total iron were detected in A549 cells with stably knockdown of LINC00641. **I** A pathway-based analysis of differential metabolites in A549 cells with LINC00641-knockdown or control. The differential abundance score captured the average, gross changes for metabolites in the pathway. A score of “1” indicated all measured metabolites in the pathway increase, and “−1” indicated all measured metabolites in a pathway decrease. **J** Relative cell viability treated with AA and IFN-γ in A549 cells for 3 days. Cell viability were detected by CCK-8 assays. #*P* > 0.05; *P < 0.05; **P < 0.01.

EMT not only contributes to tumorigenesis and metastasis, but also confers cancer cells the resistance to chemotherapy and immunotherapy [34]. Our results have shown that LINC00641 knockdown promoted EMT in the tumor cells, suggesting lung cancer patients with low LINC00641 expression might be likely to progress into a refractory cancer. Therefore, new therapeutic strategies were needed for LINC00641 low expression patients. Interestingly, KEGG (Kyoto encyclopedia of genes and genomes) pathway analysis of our RNA-seq data also revealed ferroptosis as one of the most affected cellular processes in LINC00641 knockdown cells (Fig. 6A). Recent research has suggested that cancer cells undergoing EMT were vulnerable to ferroptosis [21]. We also found that the expression of mesenchymal markers (*CDH2* and *VIM*) was negatively correlated with the resistance to ferroptosis inducers, including ML162, ML210, and RSL3; while the epithelial marker *CDH1* was positively correlated with the resistance to these ferroptosis inducers by using Cancer Therapeutics Response Portal [35] (Fig. 6B and Supplementary Fig. S5A, B). To investigate whether mesenchymal lung cancer cells were more sensitive to ferroptosis, we utilized flow cytometry to isolate cells with low E-cadherin and high N-cadherin level (referred to as A549-E^low^N^high^) and cells with high E-cadherin and low N-cadherin level (referred to as A549-E^high^N^low^) (Supplementary Fig. S5C, D). Consistent with our expectations, A549-E^low^N^high^ cells displayed lower GPX4 levels and were more sensitive to RSL3-induced ferroptosis compared to A549-E^high^N^low^ cells (Supplementary Fig. S5D, E).

Indeed, A549 and H1299 cells with LINC00641 knockdown were more sensitive to RSL3-induced ferroptosis as shown by decreased IC50 curve (Fig. 6C and Supplementary Fig. S5F). Similar results were found when LINC00641 knockdown A549 and H1299 cells were treated with Erastin, another ferroptosis inducer (Fig. 6D and Supplementary Fig. S5G). As ferroptosis level can be indicated by enhanced *PTGS2* mRNA expression and reduced SLC7A11 level [36, 37], LINC00641 knockdown up-regulated *PTGS2* expression (Fig. 6E) and decreased SLC7A11 (Supplementary Fig. S5H). Furthermore, compared to the control group, knockdown of LINC00641 increased the cellular ROS levels and the intracellular concentrations of both ferrous iron (Fe^2+^) and total iron (Fe) under the treatment of RSL3 (Fig. 6F–H), suggesting that LINC00641 downregulation could increase ferroptotic sensitivity in lung cancer cells.

Considering that ferroptosis links metabolism and redox biology [38], we next conducted a mass spectrometry to detect the comprehensive metabolomic profiling of A549 cells after LINC00641 knockdown. Principal component analysis showed clear separation between LINC00641 knockdown and control cells (Supplementary Fig. S6A). 174 differential metabolites (93 upregulated and 81 downregulated) were identified between two analysis groups (fold change ≥1.2; *P*-value < 0.05) (Supplementary Fig. S6B and Supplementary Table S2). To systematically investigate the metabolic alterations associated with LINC00641 knockdown, we performed KEGG pathway-based analysis utilizing the 174 differential metabolites. By calculating the differential abundance scores, which displayed the tendency of metabolites in a pathway to be increased/decreased relative to the control [39], arachidonic acid (AA) metabolism showed an increased tendency (Fig. 6I). Recent study has highlighted AA and IFN-γ coordinately induced tumor cell ferroptosis via ACSL4, which mediated immunogenic tumor ferroptosis [40]. The increased AA metabolism indicated the sensitivity to immunogenic ferroptosis in LINC00641 knockdown cells. As expected, A549 cells with LINC00641 knockdown were more sensitive to ferroptosis induced by the combined treatment of AA and IFN-γ, and became more sensitive with increased AA concentration (Fig. 6J). Taken together, these results demonstrated that lung cancer cells with low LINC00641 expression were more likely to undergo EMT, however, low LINC00641 expression could also cause a ferroptotic vulnerability for lung cancer treatment.

## Discussion

**Fig. 7:**
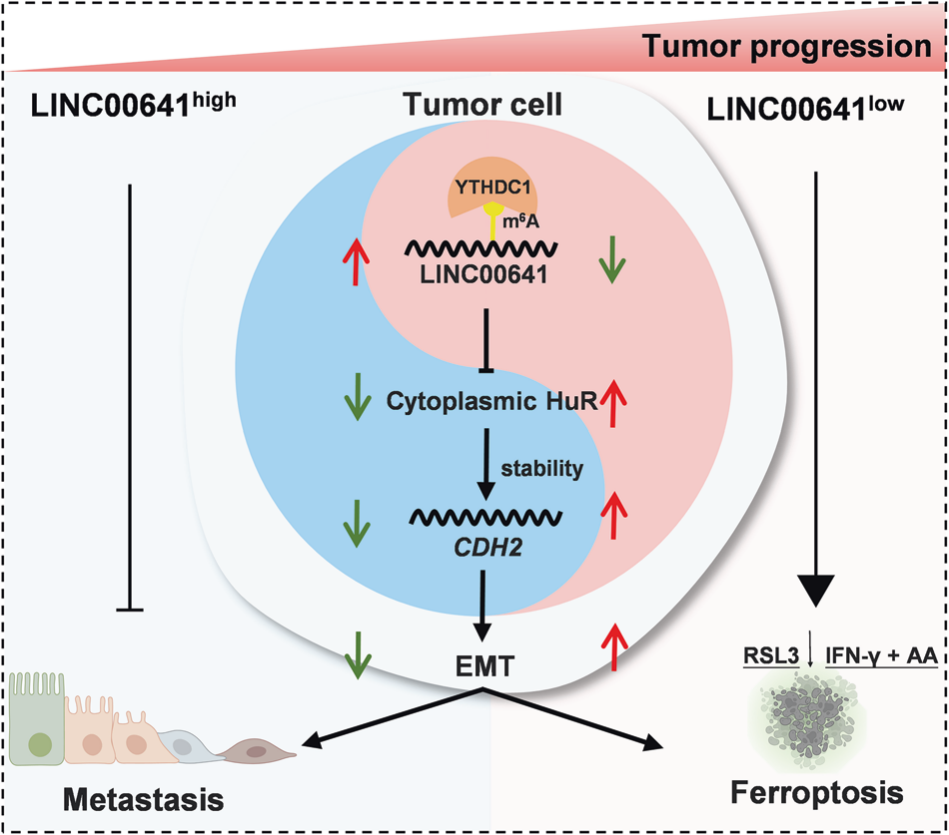
Working model. LINC00641 is modified by m^6^A and its stability is maintained by nuclear m^6^A reader YTHDC1. Knockdown of LINC00641 induces the accumulation of HuR in cytoplasm, which promotes N-cadherin by increasing the stability of *CDH2* mRNA to initiate EMT. In another aspect, knockdown of LINC00641 boosts the susceptibility to ferroptosis inducers in lung cancer cells.

Our study demonstrated that LINC00641 acted as a tumor suppressor by regulating EMT in lung cancer. LINC00641 was modified by m^6^A and the m^6^A reader YTHDC1 could maintain the stability of LINC00641. Knockdown of LINC00641 upregulated HuR protein levels (especially in the cytoplasm), which subsequently increased N-cadherin protein levels by stabilizing the *CDH2* mRNA to initiate EMT. Interestingly, knockdown of LINC00641 increased AA metabolism and boosted the susceptibility to ferroptosis inducers in lung cancer cells (Fig. 7). These findings highlighted that the LINC00641-low expression tumor harbored an EMT transition state and at the same time suffered the ferroptotic vulnerability for lung cancer treatment.

EMT has been identified as one of the major contributors to tumorigenesis and tumor progression, which confers cancer cells metastatic properties (enhancing mobility and invasion), stem cell properties, and apoptotic or therapeutic resistance [41]. During cancer progression or under apoptotic stimuli (including therapeutic) conditions, epithelial tumor cells undergo EMT that deeply alters tumor cell features (such as changes in cell polarity and intercellular junctions) and increases the expression of mesenchymal markers (such as N-cadherin and vimentin) [42, 43]. Identification of new regulatory factors in EMT could provide new strategies for tumor treatment. Recent studies have highlighted that lncRNAs as key regulators of multiple cellular process [44, 45]. Metastasis-associated lung adenocarcinoma transcript 1 (MALAT1) was the firstly identified lncRNA that contributed to the metastasis by inducing EMT in lung cancer [46]. MALAT1 could act as molecular scaffold or ceRNA to regulate the expression of target genes at the transcriptional and post-transcriptional levels [47]. Interestingly, MALAT1 could be modified by m^6^A and recognized by nuclear m^6^A reader YTHDC1 to reshape composition of nuclear speckles via liquid-liquid phase separation [48, 49]. In our study, we demonstrated low expression of LINC00641 was associated with poor outcomes in LUAD patients and knockdown of LINC00641 promoted tumorigenesis and metastasis of lung cancer. GSEA using RNA-seq data revealed that LINC00641 negatively regulated the EMT process in lung cancer cells. LINC00641, a nuclear localized lncRNA, was also modified by m^6^A and could be recognized by YTHDC1, which mediated the stability of LINC00641.

Previous studies of LINC00641 usually focused on the ceRNA mechanism, which mainly occurs in the cytoplasm. However, LINC00641 was localized primarily in the nucleus, suggesting the tumor-suppression effect of LINC00641 was not entirely dependent on the ceRNA regulatory network in the cytoplasm. To reveal the mechanisms of LINC00641 in regulating EMT, we found an RNA-binding protein HuR, which showed strong binding potential with LINC00641. Our RIP assay confirmed the binding effect of HuR to LINC00641. HuR has been involved in facilitating the EMT program and metastasis in multiple cancer types, such as gastric cancer, esophageal squamous cell carcinoma, and osteosarcoma [50]. High level of HuR protein in cytoplasm was associated with poor prognosis in lung cancer patients [31, 51]. In this study, we revealed that knockdown of LINC00641 increased the HuR protein levels (mainly in cytoplasm) in lung cancer cells. The corresponding upregulation of HuR protein mediated the pro-migration effect of LINC00641 knockdown. HuR could interact with the 3’-UTR region of a variety of mRNAs to enhance the stability of target genes [33]. In our study, we demonstrated mesenchymal marker *CDH2* as a target gene of LINC00641 by maintaining the stability of the *CDH2* mRNA through HuR, thus modulating EMT process. In addition, the increased cytoplasmic HuR after LINC00641 knockdown suggested that LINC00641-knockdown could facilitate HuR protein translocation from nucleus to cytosol, which may provide potential strategies for targeting HuR in lung cancer treatment.

Ferroptosis, a form of regulated cell death triggered by phospholipid peroxidation, has been proposed to be a promising alternative strategy to circumvent the therapy resistance of cancer cells [38, 52]. Studies have shown that cancer cells undergoing EMT could acquire therapeutic resistance and were intractable for treatment. However, EMT may turn into an Achilles’ heel of cancer, by causing a vulnerability for ferroptosis in cancer therapy. In support of this hypothesis, a previous study has revealed that ZEB1, a transcription factor that enhanced cancer metastasis by inducing EMT, promoted the sensitivity of cancer cells to ferroptosis inducers targeting GPX4 via regulating cellular lipid metabolism [22]. To test whether low expression of LINC00641 could lead to ferroptotic vulnerability for lung cancer, we first explored the Cancer Therapeutics Response Portal and found that the expression of *CDH2* mRNA (LINC00641 target gene) was negatively correlated with susceptibility to ferroptosis inducers ML162, ML210 and RSL3. Our RNA-seq and mass spectrometry data also supported that knockdown of LINC00641 altered the ferroptosis pathway and ferroptosis related AA metabolism in lung cancer cells. In our subsequent experiments, we confirmed that LINC00641 knockdown sensitized lung cancer cells to ferroptosis when treated with RSL3 or endogenous ferroptosis inducers (IFN-γ combined with AA [40]). These results might have important clinical implications that patients with low LINC00641 expression could be treated with ferroptosis-based therapy.

In conclusion, our study demonstrated that m^6^A modified LINC00641 was down-regulated in LUAD and acted as a tumor suppressor by inhibiting EMT. As a double-sided coin, low expression of LINC00641 also conveyed a ferroptotic vulnerability for lung cancer cells. These findings highlighted LINC00641 as a potential biomarker and therapeutic target for lung cancer.

## Supplementary information

Supplementary Figure S1-S6

Uncropped Western Blots

Supplementary Table S1

Supplementary Table S2

checklist

## Data Availability

All data needed to evaluate the conclusions are present in the paper. The uncropped western blots are shown in Supplementary Information as an ‘Uncropped Western Blots’ file.
