# Supplementary material for: Downregulation of N6-methyladenosine-modified LINC00641 promotes EMT, but provides a ferroptotic vulnerability in lung cancer
Source: Cell Death Dis. 2023 Jun 13;14(6):359. doi: 10.1038/s41419-023-05880-3 (PMC10264399; doi:10.1038/s41419-023-05880-3)
Supplement: Supplementary file 1 — Supplementary Figure S1-S6 [file 41419_2023_5880_MOESM1_ESM.pdf]

A

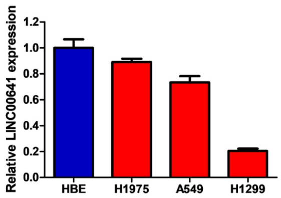

C

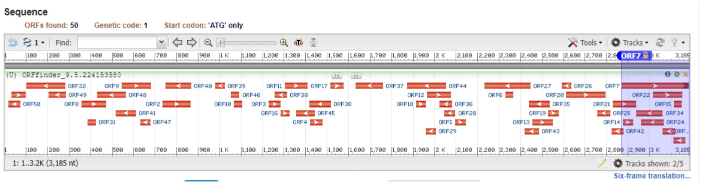

B

5'GGAGTCTGTAGTCTTCATTCAAATGTCTTGATGGCTCATTTTCCAGAAATCTCTGTAGGTCTTGAGAAGCATTGTCCTAGCTTTGGTTTATCTGAATAGTCCTATTTAAACTTGTCCCACACAGATAGCAGCCTCTTGC
TTGCCCTTCTGTTTGTACACTTGTCTATTTTCTCTTTCTTTAAATGCCTTTTGGGTGTTAATACTTCATTGGTGGTTCCATAGCCTTGTCCCCCTCTCTTTTGCAACTTGAAGGCAATTCTCAGTTCTCTTCTTGAGCACATT
CATACTTAGCAGCTTCTGAATATCCCACCCACAATTAAGGTACAATCTGGTCACATAACTTTTCCCTAGGAAGAAATGTGCTACTCAAGACTAATAGCCAAGGTTAAGAACGTGCATACTCCTGAATCGGATGTAA
ATGCATTAGTCTATTTGTTCTGTTTAGAAATAAAGATGGAACATATTTTATAGCTACTGTTAGTGCCCATGTGCCTTCTAAATATCTTTTGAATAGTTTTACTTCTCTCCATCTTGACAGCTACCATGCCAATCCAAGCT
TCCATCACCTTGCTCCTGGCTTACTGCAGTATCCTCCTGTCTACCCTTCATCCACTTTTATCTCTTTCCAATCACTTGTTCATACAACAGCCATATTGGGTTTTTTTCAAAACACACATATAATCAGGGCACTTCTCAACT
TAAAACCATTTCTGGCATTACTTGCCCTTAGGATAATGACAAAACTTTTTAACACAGTCCACAAGGCCAGAGATGGCCTGACCCTTGCTGATCTGTCCAGCCTCTGCTATATGTCCCCTTATTTTCTCTGTTCCAACCAC
ATTGGACTTTGTTCCTCTTACTAGTCATGCTCCCCACCTATTTGGTTCCCTAGTTAACTTCTCTTCATCCTTCGATTTCAACTTGTGTCACTTCCTCCAGAAAGCCTACCCTTTCTTTCTTAACCAGGCCGATAGGCTGTCA
AAACCAGGAATCTCTTCTTCGTAGCACTACAGTCATACTCATTGATACGTGTGATTATTAATGTGGGTTTTTTAAAAAATTAGAATGAAACTCAAGAGCAGGGACCACATCTATTACTTGTGATTAATATATTCCTAG
TGGTTAGCACAGTGTCTAGACAAATATATGTTGAATGAATAAATAGAAAAGGGAGAGGAAAAAGGGAAGGATTGACCCTCTGTGAGGCAGTCTTGCAAACAGCAGAAATGCAAAGCAAAGCTGAAGAACTAACT
ATTCAACACACACCTAGAGGAGCGTTGTAATGTCTTGTGCCTGTCTCAAATGGGTGCATTCTTATGGGTAAGTTTACTAGTCCAGCTTTCACTGTAATAGTTCACATTCCTCTCCTAGTGTCAGTCTCCAACAAATCC
CTCAGTAATTCGTAATCAGTAATCTCATGCCAAGGATGAGAGAGGGGCATTATCCAAAAAGTTGGGTGTGTCCATTTTTTTTTTGAGTCTTGCTCTGTTGCCCAAGCTGGAGTCCAGTGGCATGGTCTTGGCTTACTGC
AACCTCCGCCTCCCAGGTTCAAGTAATTCTGCCTCAGCCTCCTGAGTAGCTGGGATTACAGGCGCCTGCTACCTCGCCCGGCTTTTTTGTTGTTGTTATTTTTAGTAGAGACGGTGTTTCACCATGTTGGTCAGGCTGGT
CATGAACTTGTGACCTTGTGATCCGCCCGCCTTGGCCTTCCAAAGTGCTGGGATTACAGGCGTGAGCCACTGTGCCCGGCTGTTCATCTGTTTTTCAATACCTTTAAGTTCAGTTTCCCTTACAAAGCCCACTTTTTTCT
TAGTGGGAATCTCTTGTACCACTTAACACCTACATTCCTTAATTTTTAAAGTTAAAACACTTATATACTATAAAATGGACACATCTTAAGCATACAGCTTGATGAAGATTTACAAAGTAAACATGCCAGTTGAAACAC
CACTCTGATCAGGATGTGGAACATAACCAGCACTCCAGAAATCTCTCTGCCATCATTACCACCACTGGTTATCCTAGCCACTCTTCTGACTTGTATCACCATAGATAGGTTTTACTTGTTTTGAAGCCATCTATAAATG
GAATCATGGAAGTTTGGGCTTCCCTGAGAGCAGATCTTCAGAGAAAAAAAAACTTGAGTGCAGTAGTTTATTTGGCGTTTGGCTCCAGGATGCAGGAGTGGGAAAGATGGTAGAGAGACAGAAGAAAAATCAATAG
AAAGGTATTACGGAGGTTGGATATATAATGCTTCCCAGAATTGTCTGACTGAACAGGAGGCTGAAATATTTATCCGCTGGCTCCTGTTTCCAATTGATTGAGGGATGCCCCCAAGGAAGTTAACTTCACTGCAATTCT
GAGCTGTAATTGACTTTAATAAGCTCCAACAGCACTGAAAGACGTTTGGATTATTTTCAGTTTGGGATAATACTACTATGAACATTCCTGTACATGTCTTTTGAGAACACGCATGTGTACATTTGTGATGGGTATGTA
ATGGGGTGGAATTACTGGTTCATAGAATATGTGCATATTCACCTTTAGATATAGAGGCTGCCAACCATTTTTCTGAAGTGATTATACTAATTTATATTCTCACTATCTGTAAATGAACATTTCTGTTGCTCCACATCCTT
GCTGGCACTTGGTATTGATTGATTTCTCTTTTAATTTTAGGCTTTTTAATACGTGTGTAGTGATATCTCACTTGCTGTTTGTTTGCTTTTTGGTAAATTTTTTATTGAAGTATAATTTACATACAGAAAATCACACAAATT
GTGTACAGGTGTGATTTTTCACACCGTGAATACATGCAAAAACTAGTATGAAATTAAGAGTTGGAACATCACCAGTACTCCAGAAGCCCCATGCATGTTCTCTTCCAGTTACTATACCACTATCCCAAGGATGGTCTA
TTAATTCAACAGCATAGATTTATTTTGCCTGTTTTTCAACTTCAAATAAATGAAATAATATGCACTCTTGTGTCTGGCTTCTTTGACTCAACAGTATGTCTATACAACTCATCTGTATTGTTGCATGTTGTAGTTCGTTCA
TTCATACTGTTGTATACAGTATTGAATTGTATAAATTACTCATTCTGTTACTATGCATTTGGGCTGTTTCCAGTTTTGGCTATTATGAATAAAGCTGCTATGAACATCCAAAAAAAAAAAAAAAA3'

Supplementary Figure S1. LINC00641 expression is down-regulated in lung cancer cell lines and its characterization.

(A) qRT-PCR of LINC00641 expression in lung cancer cell lines (H1299, H1975 and A549) compared to that in normal human bronchial epithelial cell line (HBE). **(B)** The full sequence of LINC00641 (3185 nt). **(C)** ORFs that might code peptides of 11 to 103 amino acids were presented in the LINC00641 sequence.

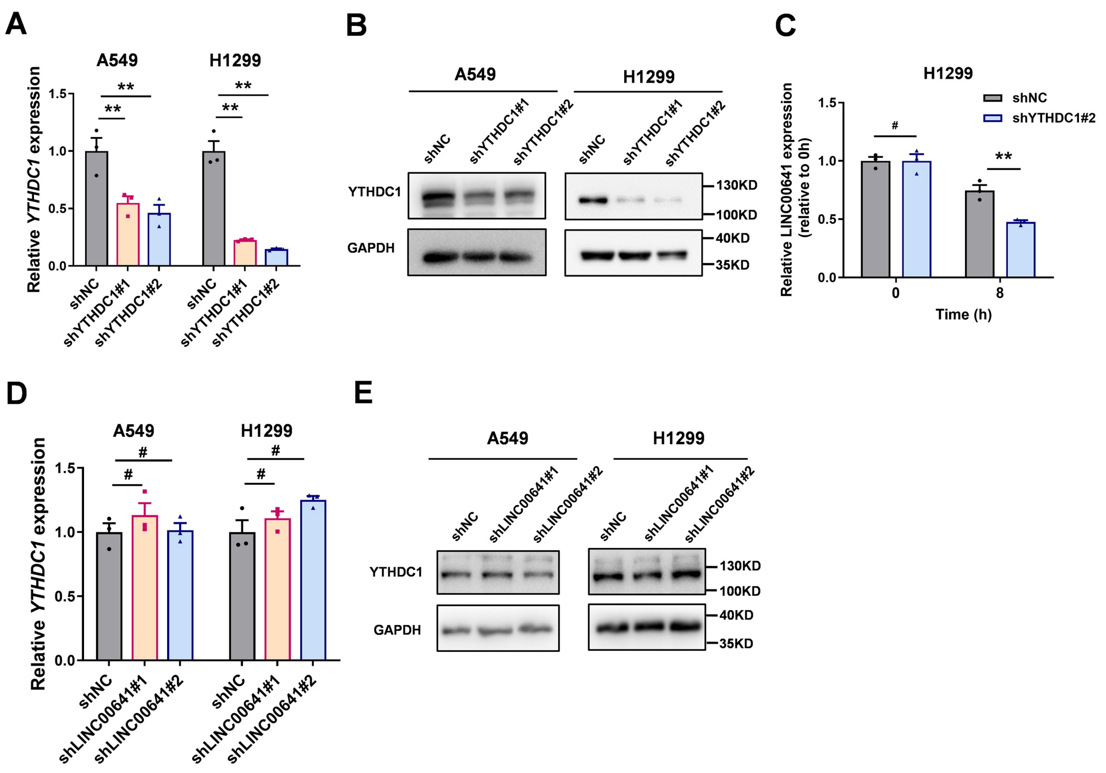

Supplementary Figure S2. The relationship between m^{6}A reader YTHDC1 and LINC00641. (A) The knockdown effects of *YTHDC1* by shRNA were detected by qRT-PCR in A549 and H1299 cells. **(B)** The knockdown effects of YTHDC1 by shRNA were detected by WB in A549 and H1299 cells. **(C)** Stability of LINC00641 was measured by qRT-PCR relative to time 0 h after blocking new RNA synthesis with Actinomycin D (5 μg/mL) in H1299 cells with stable knockdown of YTHDC1. **(D)** The expression of *YTHDC1* mRNA were detected by qRT-PCR in A549 and H1299 cells after knockdown of LINC00641. **(E)** The protein levels of YTHDC1 were detected by WB in A549 and H1299 cells after knockdown of LINC00641. #, $P > 0.05$; **, $P < 0.01$.

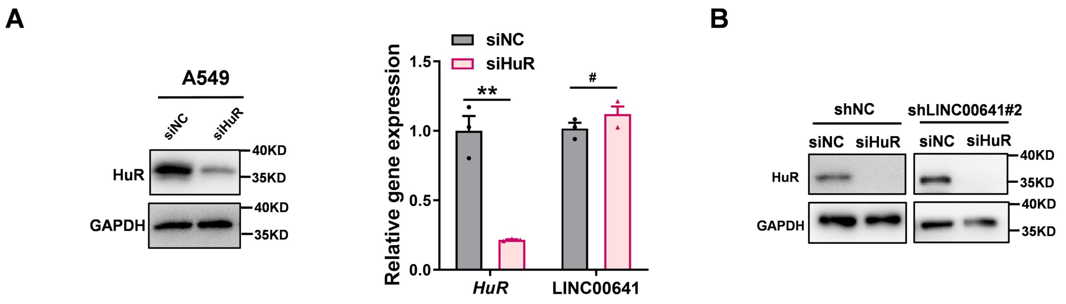

Supplementary Figure S3. The correlation between HuR and LINC00641. (A) Left: The knockdown effects of HuR protein by siRNA were detected by WB in A549 cells. Right: The expression of *HuR* mRNA and LINC00641 were detected by qRT-PCR after knockdown of *HuR*. **(B)** The knockdown effects of HuR by siRNA were detected by WB in LINC00641-knockdown A549 cells. #, $P > 0.05$; **, $P < 0.01$.

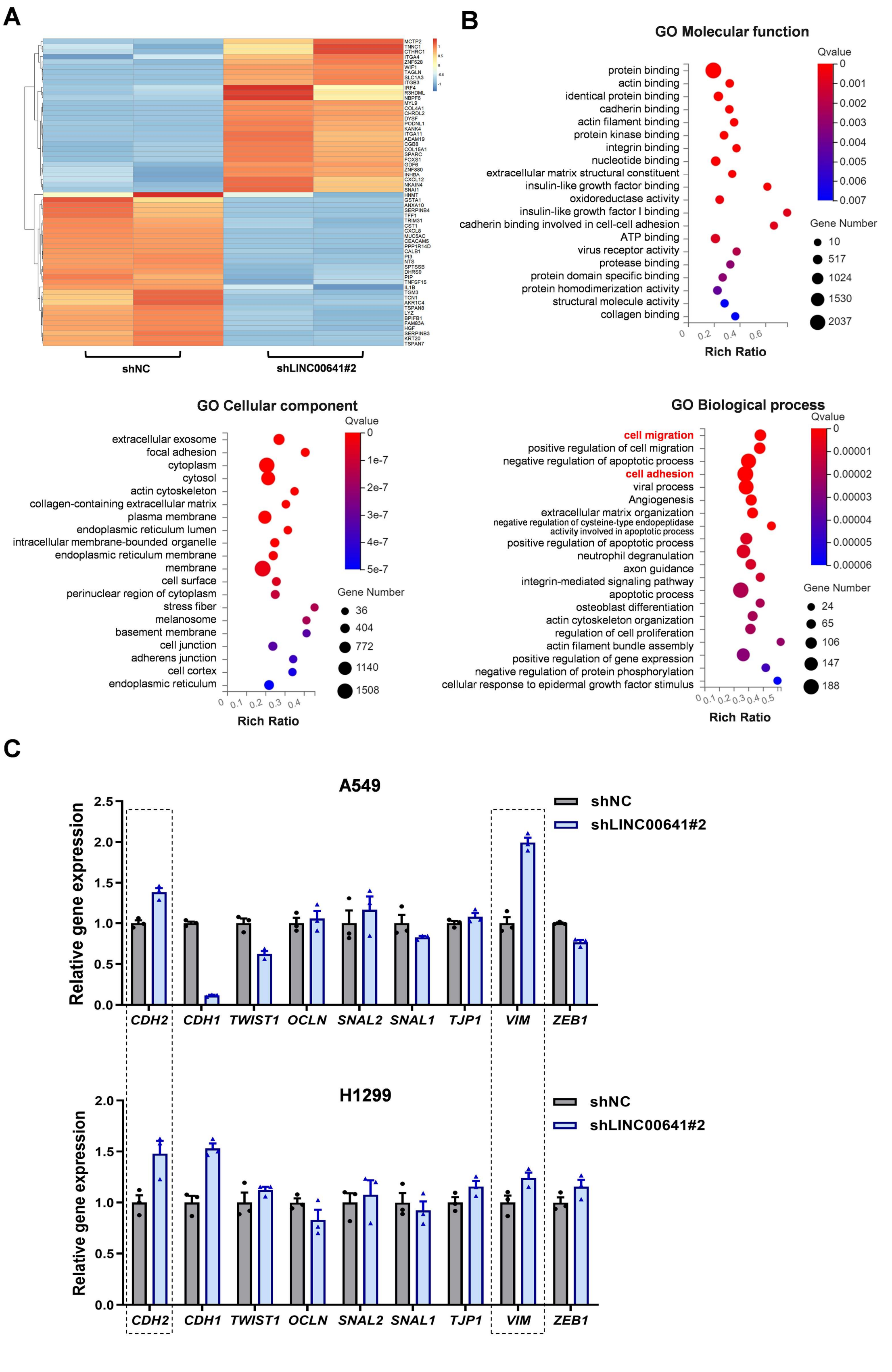

Supplementary Figure S4. LINC00641 regulates EMT process. (A) The heatmap

presented the top 30 upregulated/downregulated genes between LINC00641 knockdown and control. **(B)** Gene Ontology (GO) analysis showed that the dysregulated genes were enriched in cell adhesion and cell migration in LINC00641 knockdown cells compared with control cells in A549 cells. **(C)** The expression of EMT related genes were detected by qRT-PCR in A549 and H1299 cells after knockdown of LINC00641.

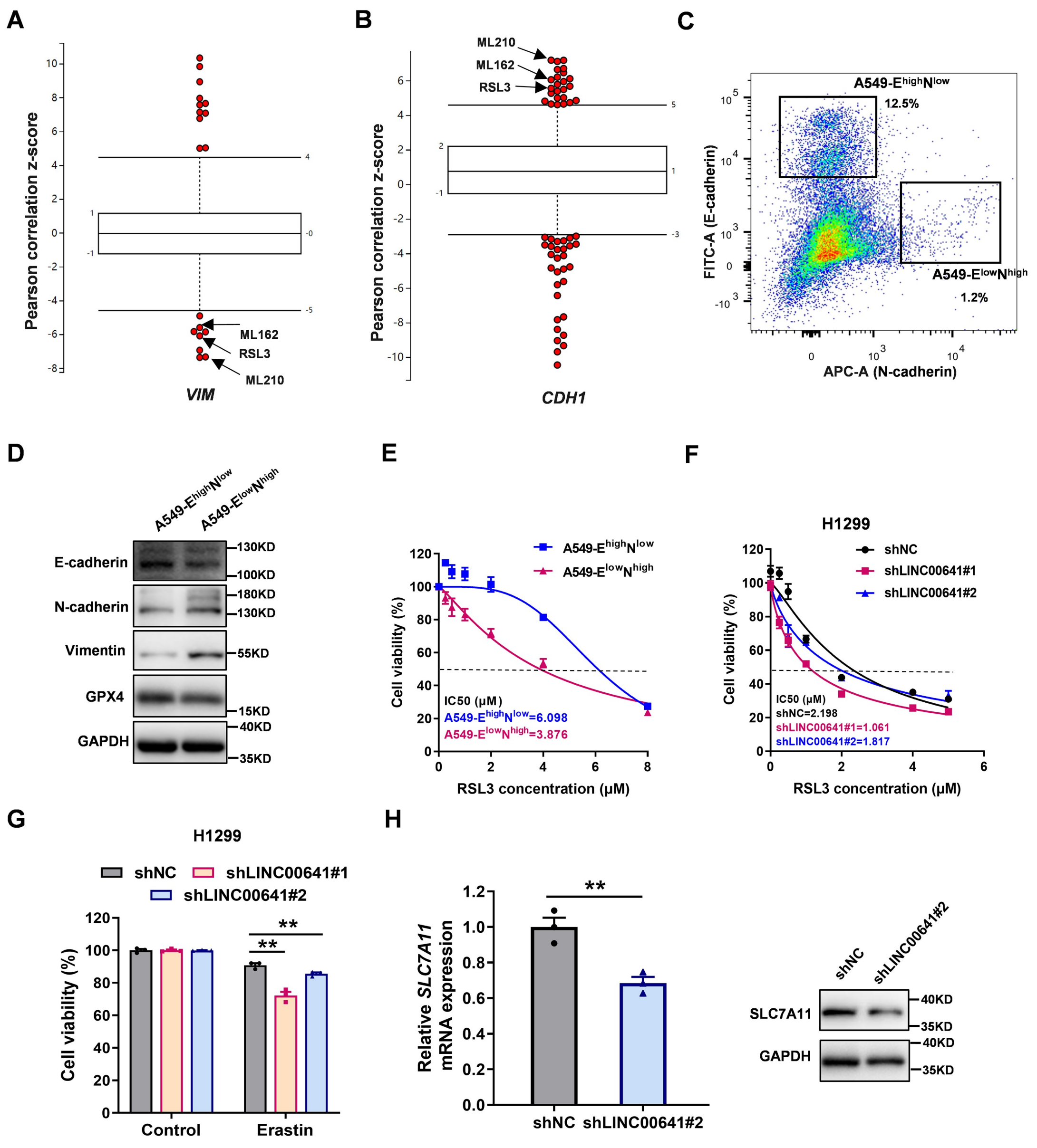

Supplementary Figure S5. The correlation of EMT markers and drug susceptibility. (A) The expression of mesenchymal marker *VIM* was negatively correlated with the resistance to ferroptosis inducer, including ML162, ML210 and RSL3. **(B)** The epithelial marker *CDH1* was positively correlated with the resistance to these ferroptosis inducers, including ML162, ML210 and RSL3. **(C)** A549-E^{low}N^{high} and A549-E^{high}N^{low} cells were sorted by using flow cytometry. **(D)** The protein levels of E-cadherin, N-cadherin, vimentin and GPX4 were detected by WB in

A549-$E^{low}N^{high}$ and A549-$E^{high}N^{low}$ cells. **(E)** RSL3 treatment was conducted to clarify the susceptibility to ferroptosis in A549-$E^{low}N^{high}$ and A549-$E^{high}N^{low}$ cells. Cell viability were detected by CCK-8 assays and IC50 curves were generated using Graphpad Prism 8.0. **(F)** RSL3 treatment was conducted to clarify the effect of LINC00641 on the ferroptosis in H1299 cells for 3 days. Cell viability were detected by CCK-8 assays and IC50 curves were generated using Graphpad Prism 8.0. **(G)** CCK-8 assays were conducted to clarify the effect of LINC00641 on the Erastin (0.5 μM)-induced ferroptosis in H1299. **(H)** The levels of SLC7A11 were detected by qRT-PCR and WB in A549 cells after knockdown of LINC00641.

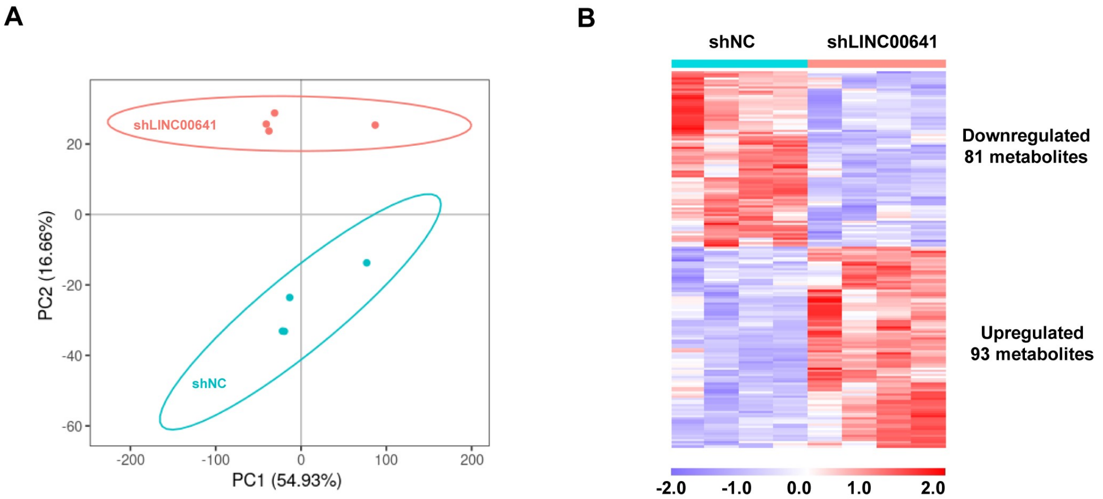

Supplementary Figure S6. Differential metabolites in A549 cells with LINC00641-knockdown or control. (A) The metabolic relationship between the control and LINC00641-knockdown A549 cells in principal component analysis. **(B)** The heat map shows the differential metabolites (93 upregulated and 81 downregulated) induced by stably knockdown of LINC00641 in A549 cells.
