# Supplementary material for: Downregulation of N6-methyladenosine-modified LINC00641 promotes EMT, but provides a ferroptotic vulnerability in lung cancer
Source: Cell Death Dis. 2023 Jun 13;14(6):359. doi: 10.1038/s41419-023-05880-3 (PMC10264399; doi:10.1038/s41419-023-05880-3)

Figure 4C

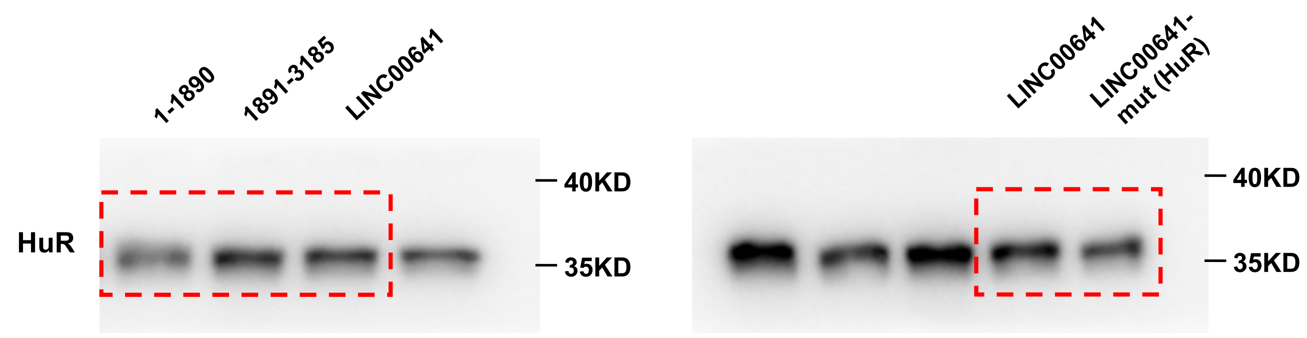

Figure 4D

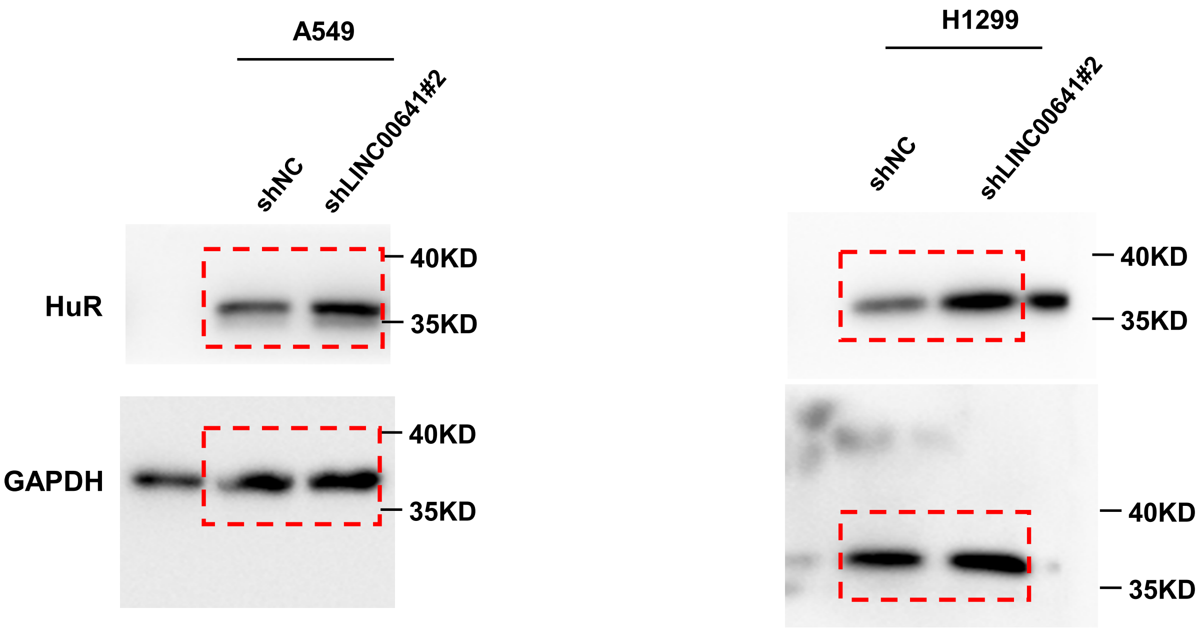

Figure 4E

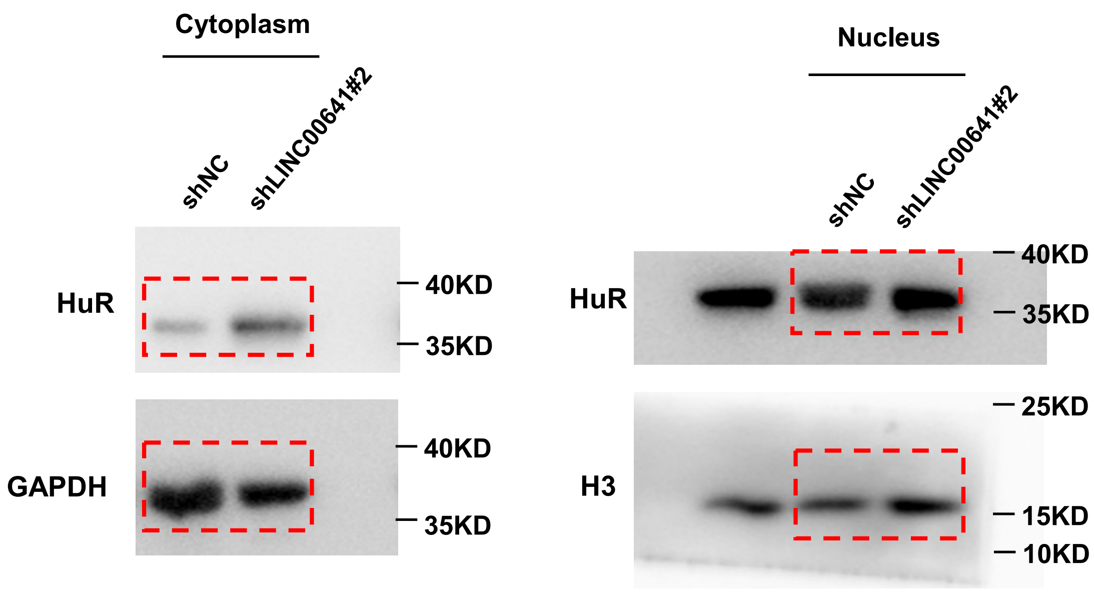

Figure 5D

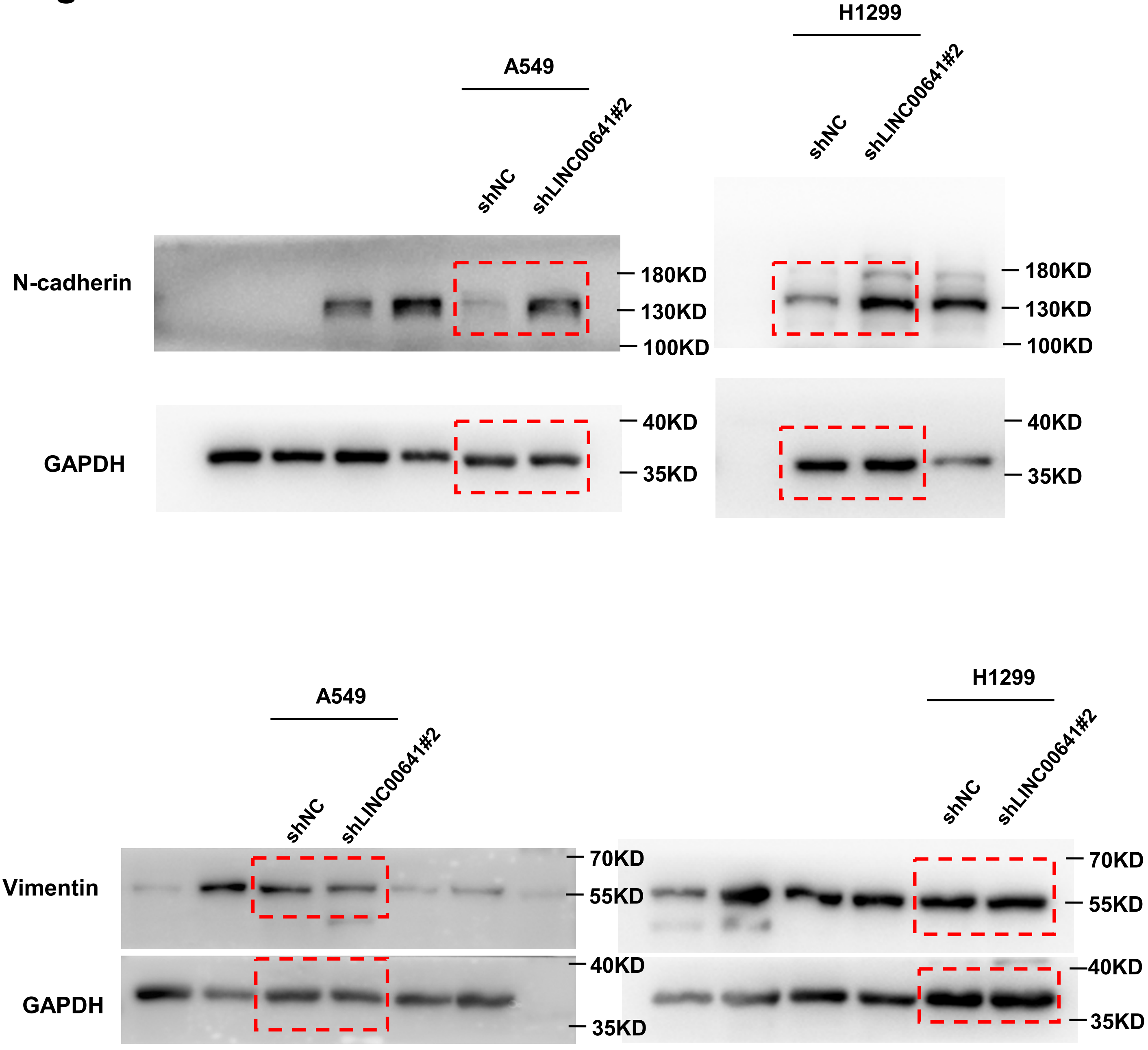

Figure 5H

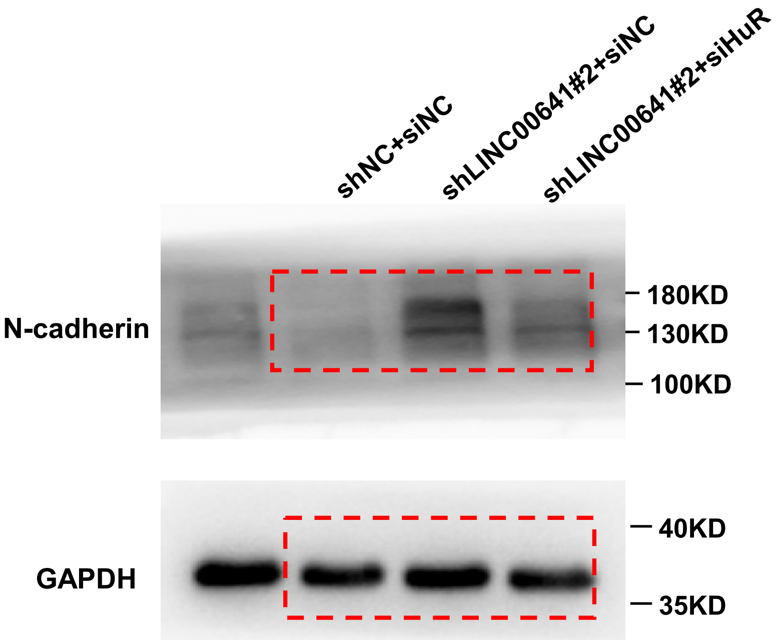

Supplementary Figure S2B

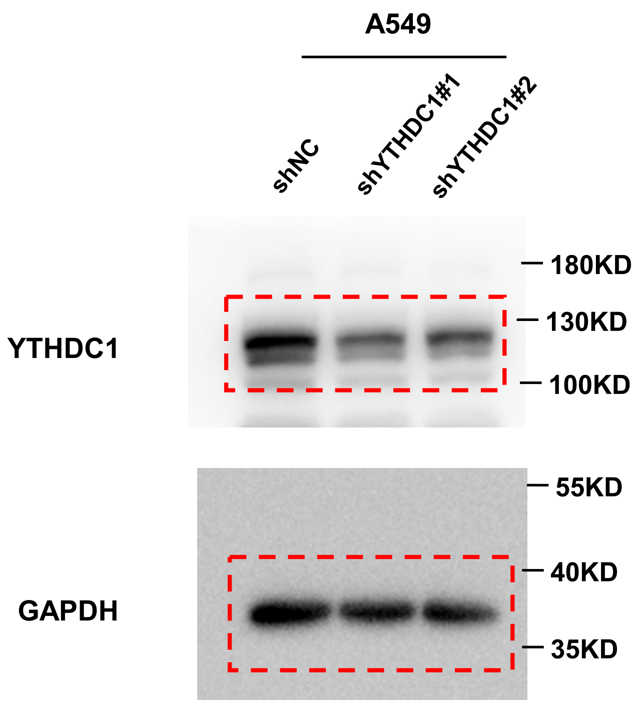

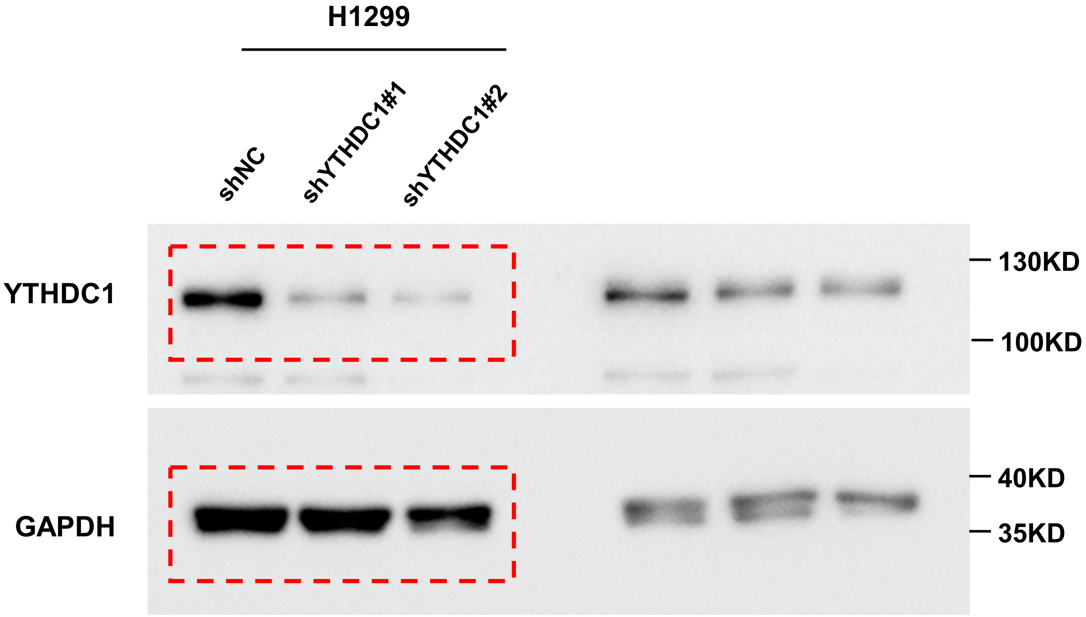

Supplementary Figure S2E

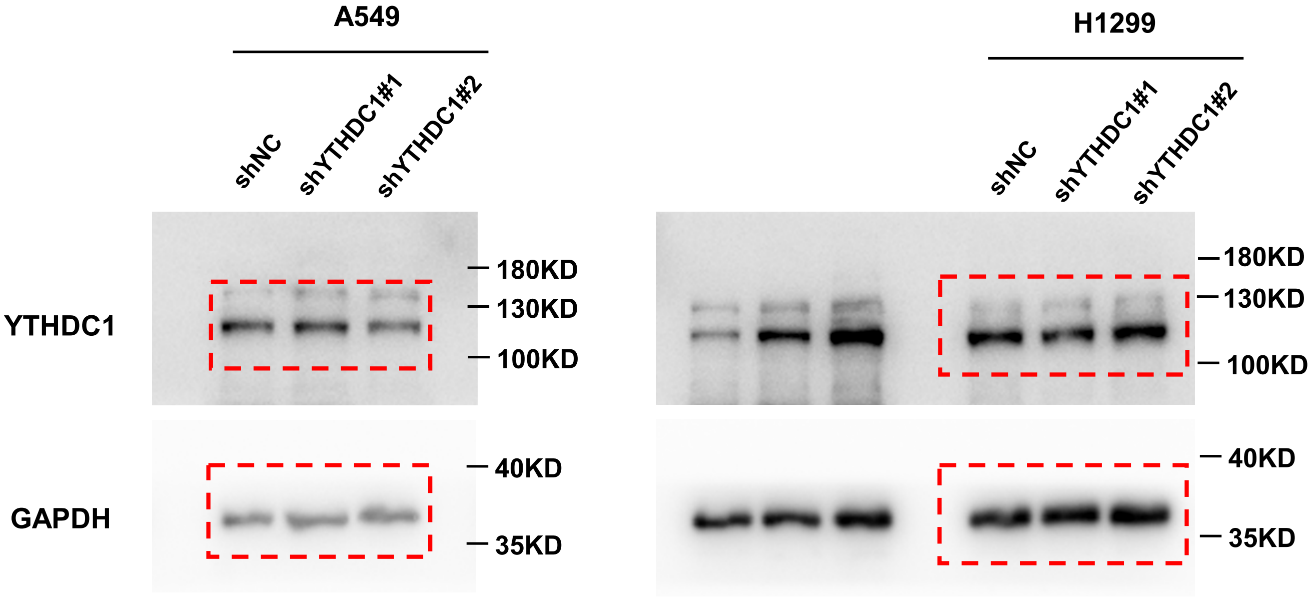

Supplementary Figure S3A

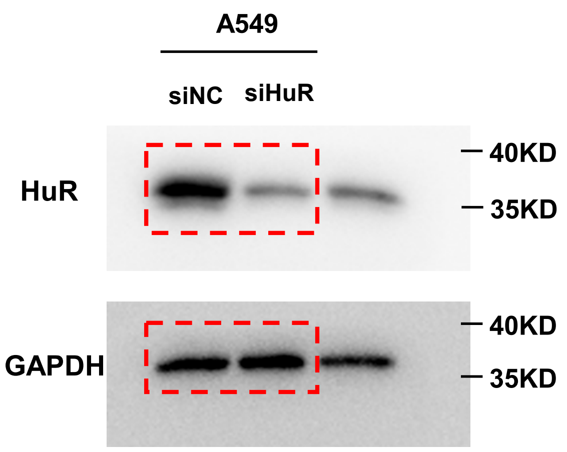

Supplementary Figure S3B

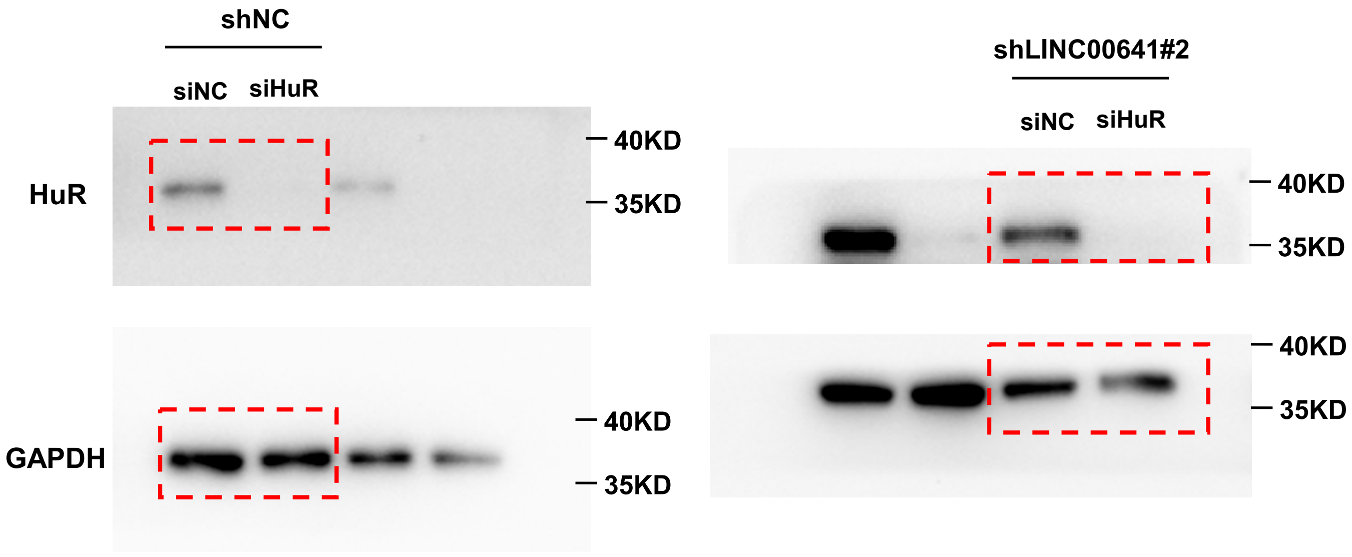

Supplementary Figure S5D

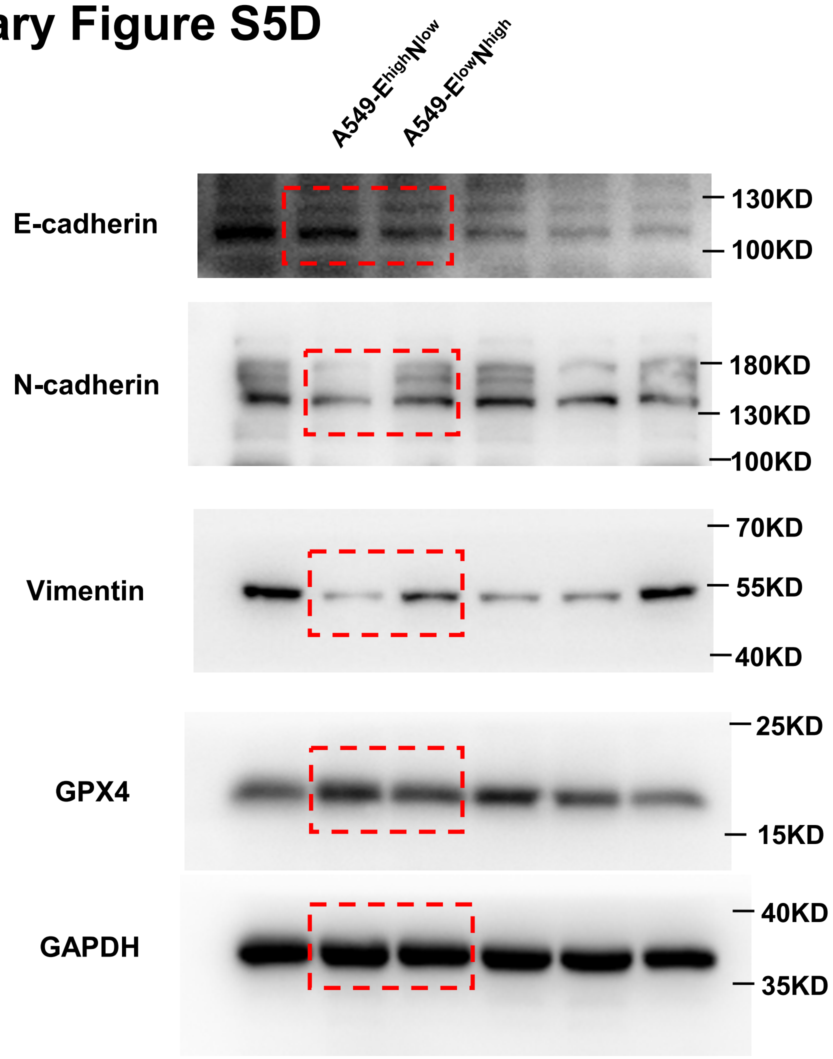

Supplementary Figure S5H

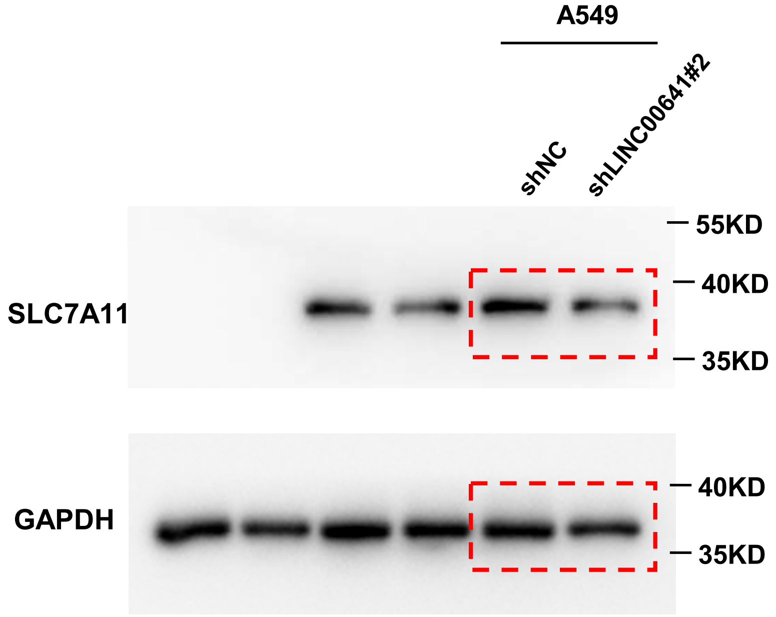

Supplement: Supplementary file 2 — Uncropped Western Blots [file 41419_2023_5880_MOESM2_ESM.pdf]
